# Thyroid Inconveniences With Vaccination Against SARS-CoV-2: The Size of the Matter. A Systematic Review

**DOI:** 10.3389/fendo.2022.900964

**Published:** 2022-06-23

**Authors:** Verdiana Caironi, Fabián Pitoia, Pierpaolo Trimboli

**Affiliations:** ^1^ Clinic for Internal Medicine, Lugano Regional Hospital, Ente Ospedaliero Cantonale, Lugano, Switzerland; ^2^ Division of Endocrinology, Hospital de Clínicas José de San Martin, University of Buenos Aires, Buenos Aires, Argentina; ^3^ Clinic for Endocrinology and Diabetology, Lugano Regional Hospital, Ente Ospedaliero Cantonale, Lugano, Switzerland; ^4^ Faculty of Biomedical Sciences, Università della Svizzera Italiana, Lugano, Switzerland

**Keywords:** thyroid, side-effects, vaccine, SARS-CoV-2, subacute thyroiditis (SAT), Graves’ disease (GD)

## Abstract

After the beginning of COVID-19 vaccination campaigns, several reports of thyroid disease possibly related to the COVID-19 vaccination progressively appeared in the literature, raising the question of whether the thyroid disorder might be a SARS-CoV-2 vaccine complication. The aim of this study was to analyze the data about COVID-19 vaccination and thyroid disease, evaluate the size and quality of related literature, assess the type of these events, and investigate their timing of onset with respect the vaccination. Pubmed/MEDLINE and Cochrane were systematically reviewed until February 2022 to retrieve the largest number of original papers, case reports, and case series articles reporting thyroid disease after SARS-CoV-2 vaccination. Forty-six articles were included with a total of 99 patients aged from 26 to 73 years were described, of whom 74.75% female. Regarding the vaccination received, 49.49% of patients received Comirnaty (Pfizer/BioNTech), 14.14% CoronaVac (Sinovac), 12.12% Vaxzevria (Oxford/Astrazeneca), 11.11% Spikevax (Moderna), 3.03% Ad26.COV2.S (Janssen, Johnson & Johnson), one patient Covaxin (Bharat Biotech) and one patient Convidecia (Cansino). In 7 cases the thyroid disorder developed after the third dose with a combination of different vaccines. Regarding the type of thyroid disorder, 59 were subacute thyroiditis (SAT), 29 Graves’ disease (GD), 2 co-occurrence of SAT and GD, 6 painless thyroiditis (PT), and single cases of thyroid eye disease and hypothyroidism associated with mixedema. The timeline between vaccination and thyroid disorder ranged between 0.5 to 60 days, with an average of 10.96 days. Considering the limited follow-up time, a complete remission was reported in most of SAT and PT cases while a persistence was observed in GD. In conclusion, both size and quality of published data about thyroid inconveniences after COVID-19 vaccination are limited; thyroid disorders may occur within 2 months after COVID-19 vaccination; among all thyroid diseases after COVID-19 vaccination, GD and SAT seem to be more frequent.

## Introduction

Since the World Health Organization (WHO) declared COVID-19 a pandemic on 11 March 2020 up till January 2022, the numbers of this infection showed an impressive increase, with more than 380 million cases, 5.7 million deaths worldwide and a total number of hospitalizations of over 280 000 in the USA (1).

The effect of COVID-19 on the Health Service has been enormous, with frequent interruptions of most routine care. The COVID-19 pandemic has triggered also one of the worst job crises since the Great Depression (2).

Measures such as physical distancing, the use of masks, and contact tracing have helped limit the transmission where they have been rigorously applied but were not sufficient to achieve a radical control of the disease. Programs promoting the clinical development of vaccines have been established to permit the reduction of the severe consequences of COVID-19, allowing the earliest possible stabilization of health care systems, communities, and economies (3).

At the time of writing, there are 181 vaccine candidates, 604 vaccine Trials, and 72 countries with vaccine Trials. There are at this time 44 vaccines in phase I, 64 in phase 2, 65 in phase III and 33 approved (4). They are developed with different methods such as protein subunits, virus-like particles (VLP), inactivated virus, DNA-based vaccine, RNA-based vaccine, non-replicating and replicating viral vectors, and live-attenuated viruses (4, 5).

The vaccines approved to date are highly effective against severe disease and death (6). It is reassuring that although vaccine effectiveness against infection appears to decline with increasing time since vaccination, it continues to perform well against severe disease and death (7).

Since the beginning of COVID-19 vaccination campaigns, several types of side effects have been described; the most common side effects were pain, redness or swelling at the site of injection, tiredness, headaches, chills, muscle/joint aches and fever predominantly in younger population (8–10).

Among the possible SARS-CoV-2 vaccine complications, thyroid disease was not initially described, but early on, case reports of subacute thyroiditis, Graves’ disease, and thyroid eye disease possible related to the COVID-19 vaccination began to appear in the literature, and the question whether the thyroid disorder might be a SARS-CoV-2 vaccine complication has been raised. Subsequently, several case reports, case series, letters to editors, and reviews were published on the thyroid sequelae experienced by patients after receiving COVID-19 vaccine.

The present study seeks to analyze and summarize data about COVID-19 vaccination and thyroid disease. The literature was systematically reviewed to retrieve the largest number of original papers, case reports, and case series articles reporting thyroid disease in patients vaccinated against SARS-CoV-2. Therefore, this study was undertaken to 1) evaluate the size and quality of the literature about thyroid inconveniences and side-effects of vaccine against the COVID-19, 2) evaluate the type of these events, and 3) analyze the timing of onset of these events with respect the vaccination.

## Methods

### Conduction of Review

The systematic review was performed according to the PRISMA statement (11).

### Search Strategy

A specific search strategy was planned; 1) sentinel studies were searched in PubMed; 2) keywords and MeSH terms were identified; 3) PubMed and Cochrane databases were searched for MeSH/terms identified [i.e., thyroid* OR hypothyroid* OR hyperthyroid* AND (SARSCov* OR COVID) and vaccin*]; 4) studies reporting the occurrence of thyroid inconveniences and/or side effects with vaccination against SARS-CoV-2 were detected; 5) references of included studies were finally screened to retrieve further papers. Studies with overlapping data were excluded. The initial search was performed on 5 January 2022 and the last search on 16 February 2022. Articles written in English were always included while papers in other languages were included appropriately. No publication year restriction was applied. Two investigators (VC, PT) independently searched papers, screened them, reviewed their full-texts, and selected those meeting the inclusion criteria.

### Data Extraction

The following information were independently searched and extracted by two authors (VC, PT) from the included studies: general information (authors and their country of origin, journal, year of publication, study type), patient data (gender, co-morbidity, family history, preexisting thyroid disease), thyroid effects by vaccination (thyroid diagnosis, symptoms and signs, physical examination, thyroid laboratory tests, ultrasound presentation, scintiscan findings, treatment, long-term follow up), and vaccination features (vaccine dose, days until symptoms). Findings were cross-checked between the same two authors and discordances were mutually discussed with a third one. The risk of bias of included studies was assessed independently by two authors (VC, PT) using a Joanna Brigg Institute (JBI) critical appraisal tool (12).

## Results

### Articles Retrieves

**Figure 1:**
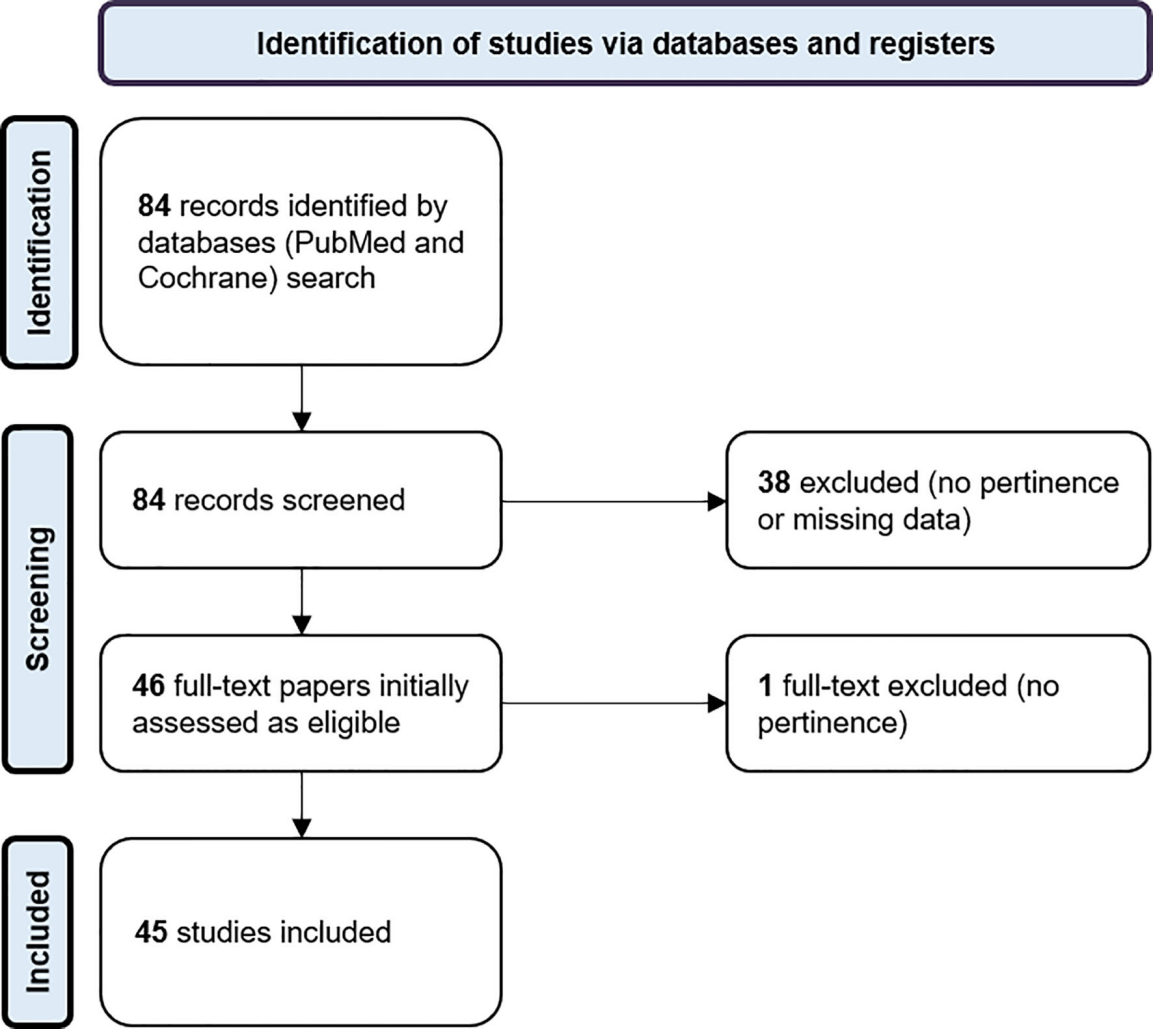
Flow of records.

According to the above-mentioned search strategy, 84 articles were initially found; among these 39 articles were excluded and 45 were finally included (13–57). **Figure 1** reports the flow of records.

### General Articles Information

The data are summarized in the **Supplementary Tables**. Thirty-two articles were published in 2021 and 14 in 2022; 24 were case reports, 16 case series and 5 letters to the editor. Among the cases reported, 8 were from USA, 6 from Turkey, 4 from Spain, 4 from Italy, 3 from Greece, 2 from Japan, 2 from South Korea, and 2 from UK. The authors’ country affiliation for the other articles was Iran, Belgium, Austria, Norway, Australia, Brazil, Mexico, India, Cyprus, Thailand, China and Ireland.

### Patient’s Features (Personal History, Previous Thyroid Disease, Family History)

We collected data of 99 patients with mean age of 44.1 years (range from 26 to 73 years), of whom 74 (74.75%) female and 25 (25.25%) male.

Twenty-three patients (23.23%) had no comorbidities, 5 had non-thyroid autoimmune disease (systemic lupus erythematosus - SLE, vitiligo, undifferentiated connective tissue disease, ankylosing spondylitis, type 1 diabetes), 4 had diabetes (one type 1, one type 2 and the other two non-specified), one had pre-diabetes, 4 had hypertension and 4 had an oncologic disease (2 colorectal cancer, 1 breast cancer, 1 papillary cancer). Other single patients presented dyslipidemia, ulcerative gastritis, intraocular hypertension, endometriosis, infective disease (endometritis), obesity, asthma and insipidus diabetes. Four patients were smokers (2 active and 2 with a history of smoking). For 14 patients no other information was provided but the absence of autoimmunity and for 29 patients nothing was detailed about comorbidity.

No previous thyroid disease was present in 54 patients, while 17 (17.17%) patients were already known for a thyroid disorder: 6 had one or more thyroid nodules, 4 Graves’ disease, 4 hypothyroidism (3 of them due to Hashimoto’s disease), 3 had had subacute thyroiditis (SAT), one had had papillary cancer, and 2 had had a thyroid resection (one for a benign nodule, one for non-detailed reason). No information about previous thyroid history was available in the remaining 28 (28.28%) cases.

Information about family history was given for 58 patients (58.58%): 47 patients had no notion of thyroid or autoimmune disease, 4 had a positive family history for hypothyroidism (the origin was not specified in one case and was a Hashimoto’s disease in 3 cases), 3 reported a family history of hyperthyroidism (in one case GD was present in father, mother and brother, in another one, the mother suffered from GD, and one patient had both grandmothers with unspecified hyperthyroidism), 2 had a positive family history for other thyroid diseases (benign nodule and unknown thyroid disease), 3 reported familial cases of other autoimmune diseases (2 patients SLE and one patient Sjogren’s syndrome).

### Thyroid Diseases Developed After Vaccination

**Figure 2:**
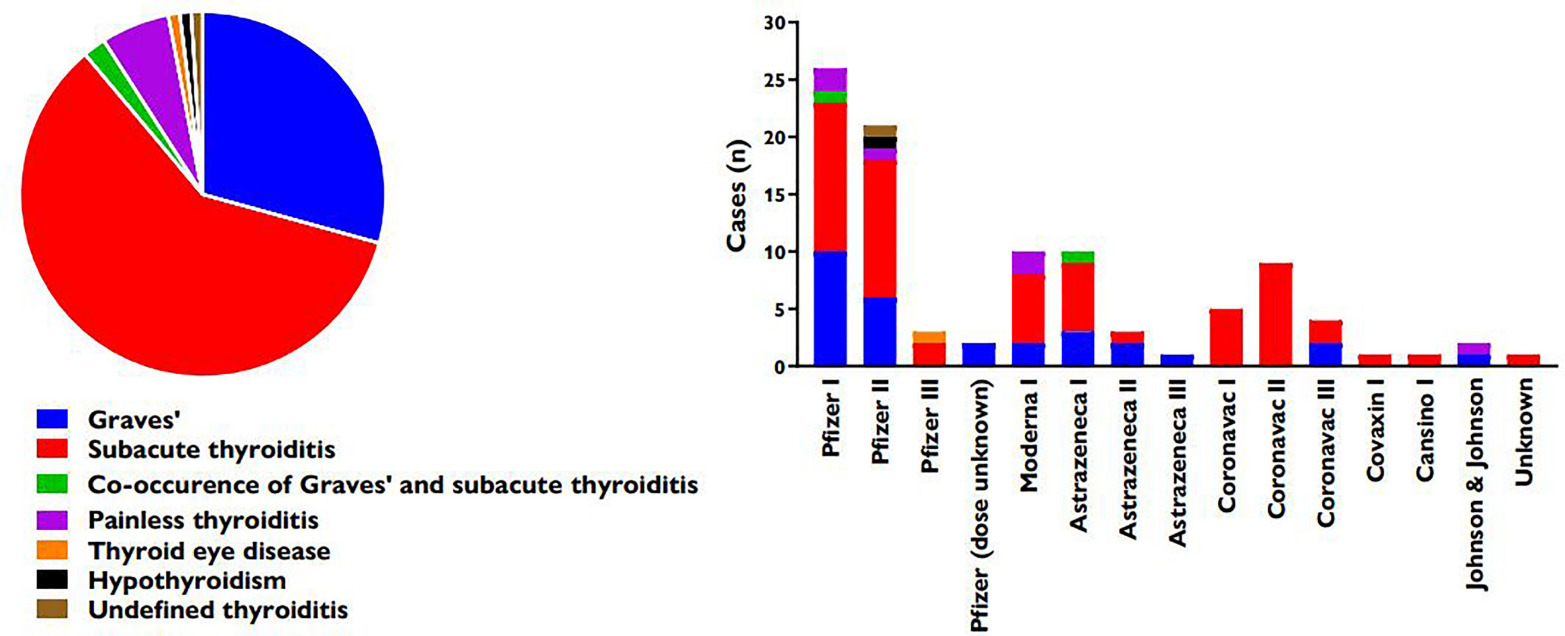
Relative prevalence of thyroid diseases recorded after vaccination (left) and distribution according to vaccine (right).

The thyroid diseases described in the analyzed literature were 59 SAT, 29 Graves’ disease (GD) (4 of which were relapses or worsening of pre-existent GD), 2 co-occurrence of SAT and GD, 6 painless thyroiditis (PT), one of which associated with thyrotoxic periodic paralysis, 1 thyroid eye disease (TED), 1 hypothyroidism associated with mixedema, and 1 case described as generic thyroiditis. **Figure 2** illustrates the relative prevalence of published cases of thyroid diseases recorded after vaccination.

### Course of Thyroid Disease

Main symptoms reported for SAT were cervical pain, palpitations, neck swelling/goiter, fever, asthenia, weight loss and sweating; there were 2 cases of asymptomatic patients. For GD the most frequent symptomatology were palpitations, weight loss, irritability, asthenia, tremor, and sweating. There was a case of a thyroid storm. PT presented with palpitations and weight loss.

Globally, information about evolution was provided for 73 cases (73.74%). Concerning GD, it is available for 16 (55.17%) of the cases. Just one case of remission after 10 weeks was described (54) but without indication on TRAb status nor on treatment administration, continuation, or interruption. A follow-up of TRAb is reported for 3 cases (10.34%): in two cases there was a persistence of TRAb positivity (44) and in one case a normalization was obtained with treatment (55). A case of thyroid eye disease after 10 weeks was also described (31). As for SAT, a follow up is available for 48 cases (81.36%). A complete remission was reported for 31 cases (64.58%). For 27 cases, information was provided about timing of resolution, with an average of 8.88 weeks (range: 4-20 weeks). There was no remission at follow-up for 17 cases (34.69%): an evolution to hypothyroidism was described for 8 cases with an average follow-up of 6.9 weeks (range: 3-12 weeks). In 7 cases of persistent disease, no further information was given about thyroid function. In the two cases of co-occurrence of GD and SAT, no follow-up information was available. Among the 6 cases of PT, the evolution was reported for 5 patients with absence of remission in only one case, characterized by hypothyroidism at 8 weeks of follow-up.

### Timeline Between Vaccination and Thyroid Event

The symptoms appeared an average of 10.96 days after the administration of the vaccine with a minimum value of 0.5 days and a maximum value of 60 days. In most cases the disease onset occurred between 0 and 15 days.

### Vaccination Features (Vaccine Type and Doses)

Forty-nine patients (49.49%) received a vaccination with Comirnaty (Pfizer/BioNTech), 25 of whom reported symptoms after the first dose and 22 after the second one; in two cases, the number of the dose was not specified. For 14 patients (14.14%), the vaccine was CoronaVac (Sinovac): 5 of them developed thyreopathy after the first dose and 9 after the second one. For 12 patients (12.12%), the vaccine was Vaxzevria (Oxford/Astrazeneca): 9 of them reported thyreopathy after the first dose and 3 after the second one. 11 patients (11.11%) were vaccinated with Spikevax (Moderna), with 10 cases of thyroid disease after the first dose and one case after the second dose. 3 patients (3.03%) received a single dose of Ad26.COV2.S (Janssen, Johnson & Johnson). One patient was vaccinated with a single dose of Covaxin (Bharat Biotech). One patient received a single dose of Convidecia (Cansino). In one case the name of the vaccine was not mentioned. In 7 cases, the patients developed thyroid symptomatology after the third dose with a combination of different vaccines: in all these cases they received 2 doses of CoronaVac, after which 5 of them had a 3rd dose of Pfizer, one of them had a 3rd dose of Vaxzevria and the last one had 2 doses of Comirnaty.

### Risk of Bias and Quality Assessment

Data extracted from the articles were almost complete in all the above outcomes except that regarding co-morbidity, previous thyroid disease, familial history, and the laboratory tests. In fact, information about co-morbidity was absent in 29 (29.29%) of cases, thyroid history was not detailed in 28 (28.28%) of patients and family history was missing in 41 (41.41%) of cases; the antibody profile was reported in 100% of GD cases (only TRABs were considered), but, as for SAT, in 21 (35.59% of total SAT cases, excluding the two cases of co-occurrence of SAT and GD) cases the TRABs were not reported, in 16 (27.12%) cases the anti-TG antibodies were missing, in 8 cases (13.56%) the anti-TPO antibodies were not specified and in 7 cases (11.86%) no antibody value was provided. This could lead to a certain risk of bias due to missing results.

According to JBI tool, after excluding case report (i.e., sample size <5), the risk of bias of 4 case series could be assessed and shown in **Table 1**. The risk of bias for each study could be judged as low in 6 of 10 items. Inclusion criteria, method used for identification of the condition, demographics of the participants, and clinical information were reported. It was not possible to assess if the inclusion of the participants was complete and inclusive because it was not reported as the statistical analysis. Follow up was mentioned in 3 out of 4 studies, being it too short to evaluate long-term complications.

**Table 1:** Study quality assessment with JBI tool: risk of bias for each case series (i.e., series including at least five cases) included in the present systematic review.

	1	2	3	4	5	6	7	8	9	10
(44)	L	L	L	NR	NR	L	L	H	L	NR
(32)	L	L	L	NR	NR	L	L	H	L	NR
(54)	L	L	L	NR	NR	L	L	H	L	NR
(39)	L	NR	NR	NR	NR	L	L	NR	L	NR

Risk of bias was defined according to the below questions, being it defined as low (L), high (H), or not reported (NR).

Questions:

1. Were there clear criteria for inclusion in the case series?

2. Was the condition measured in a standard, reliable way for all participants included in the case series?

3. Were valid methods used for identification of the condition for all participants included in the case series?

4. Did the case series have consecutive inclusion of participants?

5. Did the case series have complete inclusion of participants?

6. Was there clear reporting of the demographics of the participants in the study?

7. Was there clear reporting of clinical information of the participants?

8. Were the outcomes or follow-up results of cases clearly reported?

9. Was there clear reporting of the presenting sites’/clinics’ demographic information?

10. Was statistical analysis appropriate?

## Discussion

Since the beginning of 2021, after the introduction and wide-ranging administration of SARS-CoV-2 vaccination, an increasing number of reports were published, suggesting a possible association between thyroid dysfunctions and SARS-CoV-2 vaccines. We hereby conceived a systematic review to explore the relationship between thyroid disorders and SARS-CoV-2 vaccination.

The first aim of our review was to better clarify the size of the matter about thyroid side effects with COVID-19 vaccination. It is important to emphasize that, till 6 March 2022, a total of 10,704,043,684 vaccine doses have been administered (58). Considering this high number of vaccine doses given worldwide, it was found that the number of published cases about thyroid side-effects is very small (99 cases). A similar result was found by Ippolito et al. They performed a systematic review of the literature regarding exclusively SAT (and no other thyroid disease) after SARS-CoV-2 vaccination and found 51 patients who developed SAT (59). From a librarian point-of-view, we can conclude that the incidence of thyroid inconveniences with SARS-CoV-2 vaccination is very rare and negligible. Nevertheless, an underestimation of these data must be considered, as usual in any systematic review of published articles. In fact, this incidence may be higher than that found in the online medicine databases since most physicians do not report (all) cases, many cases go untreated, not everyone gets access to medical/endocrine clinic, there are also ignored cases, etc. All in all, the paucity of published cases should prompt us to conclude that the benefits of SARS-CoV-2 vaccination overweigh the thyroid adverse effects.

The second aim of our review was to investigate the timeline between vaccination and thyroid disorder: this ranged between 0.5 to 60 days, with an average of 11 days; most of cases occurred in the first 15 days after vaccination. Most cases of thyroid disease reported were SAT or GD, with a minority of cases of PT and a half of patients received Pfizer/BioNTech vaccination. The clinical presentation of SAT appeared to be similar to “classic” forms of SAT, with cervical pain, neck swelling/goiter, fever, asthenia, and palpitations. From a biochemical point of view, the patients almost always presented with thyrotoxicosis and elevated serum inflammatory markers. When available, ultrasonography showed an enlarged thyroid gland, with heterogeneous echo-structure and hypoechoic areas, and a decreased vascularization at the Doppler. Most patients were treated with NSAIDs or prednisone (or other glucocorticoids) or a combination of both, sometimes in association with propranolol. Among the cases for which a follow-up was performed, a complete remission is reported for 64.58%, in a time comprised between 4 and 20 weeks. Even for GD, the clinical picture was characterized by a “classic” symptomatology, encompassing palpitations, weight loss, irritability, asthenia, tremor, and sweating. Biochemistry showed a thyrotoxicosis profile and ultrasonographic/scintigraphic features of these patients, when available, were consistent with classic GD. The treatment was based on thionamides (methimazole or carbimazole), frequently in association with B-blockers (propranolol or atenolol). When a follow-up was available, a persistence of disease (either controlled or not by the treatment) was reported; just one case of remission after 10 weeks was described (40) but without indication on TRAb status nor on treatment. Concerning the 6 cases of PT, symptoms reported included palpitations and weight loss. Initial biochemical presentation was a thyrotoxicosis and ultrasonography showed a gland of normal or increased dimension, with heterogeneous echostructure and normal or reduced blood flow. No treatment was introduced and, as for evolution, a remission was reported in 4 cases and the development of hypothyroidism occurred in one case.

The development of thyroid disorders (in particular SAT) in adult population following other kinds of vaccination was described in the literature. To our knowledge, 5 case reports of thyroiditis following influenza vaccination and 1 case after hepatitis B vaccination are published (60–65). Concerning Human Papillomavirus (HPV), the literature is more consistent. Several systematic reviews found an association between HPV vaccination and thyroid disease (66–70).

To explain the association between SARS-CoV-2 vaccination and thyroid disease, 3 main mechanisms have been proposed. The first one is shared by different types of vaccines against COVID-19 regardless of the presence of adjuvants in the excipients and is based on molecular mimicry between COVID-19 viral proteins and human tissues. The immune reaction to SARS-CoV-2 Spike Protein and SARS-CoV-2 Nucleo protein leads to the production of cross-reactive antibodies and their interaction with different tissue antigens, including thyroid tissue, may be associated with autoimmune disorders (71). The second mechanism that could be involved is bystander activation. It is an antigen non-specific mechanism in which an infection or a vaccination causes a stimulation of innate immunity and finally leads to the activation of autoreactive T cells (72). Indeed, bystander activation is one of possible pathogenetic mechanisms evoked in autoimmune thyroiditis and Graves’ disease (73).

The third mechanism, one of the most frequently repeated postulations in the discussion of different case reports, is linked to the use of adjuvants in vaccine excipients. Adjuvants such as aluminum-based salts, Toll-like receptor (TLR) agonists, emulsions, and other novel adjuvants are critical components of vaccines. They have distinctive physicochemical properties, which can be significant in regulating the strength, duration, and types of immune responses. Furthermore, a hypothetical autoimmune disorder called Autoimmune/autoinflammatory Syndrome Induced by Adjuvants (ASIA) has been proposed to explain autoimmune disorders after vaccination (74, 75). However, thyroid disease, as other autoimmune diseases, have a complex multifactorial etiology and many factors can contribute to their onset. The exact pathogenetic mechanism that explains the causal link between thyroid disease and vaccination is not yet fully understood and they are also difficult to study, so further data are needed to establish this association with certitude (76).

A more recent study by Paschou et al. was published after the date of our last search and reported interesting data in this field. In fact, it provided evidence that patient with autoimmune thyroiditis present similar immunological response to COVID-19 BNT162b2 vaccine (Comirnaty, Pfizer/BioNTech) with healthy subjects. This vaccination may affect thyroid function, namely could decrease TSH and T3 level (77).

In conclusion, the present systematic review found that 1) the size of the matter of thyroid inconveniences after COVID-19 vaccination is overall small and probably negligible, 2) thyroid disorders may occur within 2 months after COVID-19 vaccination, 3) among all thyroid inconveniences after COVID-19 vaccination, GD and SAT seem to be more frequent.

## Data Availability Statement

The original contributions presented in the study are included in the article/**Supplementary Material**. Further inquiries can be directed to the corresponding author.

## Author Contributions

VC: Data collection and curation, formal analysis, writing-original draft, writing- review and editing; FP: critical supervision; PT: conceptualization, data curation, methodology, resources, supervision, writing-original draft, writing review and editing. All authors contributed to the article and approved the submitted version.

## Conflict of Interest

The authors declare that the research was conducted in the absence of any commercial or financial relationships that could be construed as a potential conflict of interest.

## Publisher’s Note

All claims expressed in this article are solely those of the authors and do not necessarily represent those of their affiliated organizations, or those of the publisher, the editors and the reviewers. Any product that may be evaluated in this article, or claim that may be made by its manufacturer, is not guaranteed or endorsed by the publisher.
