# Supplementary material for: Thyroid Inconveniences With Vaccination Against SARS-CoV-2: The Size of the Matter. A Systematic Review
Source: Front Endocrinol (Lausanne). 2022 Jun 23;13:900964. doi: 10.3389/fendo.2022.900964 (PMC9259875; doi:10.3389/fendo.2022.900964)
Supplement: Supplementary file 1 [file DataSheet_1.zip › Supplementary Table 1.DOCX]

| **Supplemental Table 1. General features of patients** | | | | | | | | | |
| --- | --- | --- | --- | --- | --- | --- | --- | --- | --- |
| **Ref.** | **Year** | **Article type** | **Contry** | **Age** | **Gender** | **Family History** | **Co-morbidity** | **Pre-existing thyroid disease** | **Diagnosis** |
| 13 | 2021 | case report | China | 40 | F | no | NA | subclinical hypothyroidism (TPO-) | GD |
| 57 | 2021 | case series | Spain | 38 | F | NA | NA | NA | SAT |
| 57 | 2021 | case series | Spain | 32 | M | NA | Type 1 diabetes | NA | PT |
| 57 | 2021 | case series | Spain | 38 | F | NA | Schizophrenia | no | GD |
| 14 | 2021 | case report | Ireland | 42 | F | maternal hypothyroidism | NA | NA | SAT |
| 43 | 2022 | case series | Italy | 29 | F | no | no | no | PT |
| 43 | 2022 | case series | Italy | 34 | M | no | no | no | PT |
| 44 | 2022 | case series | Spain | 71 | F | no | NA | NA | GD |
| 44 | 2022 | case series | Spain | 42 | F | no | NA | NA | GD |
| 44 | 2022 | case series | Spain | 54 | F | no | NA | NA | GD |
| 44 | 2022 | case series | Spain | 46 | F | no | NA | NA | GD |
| 44 | 2022 | case series | Spain | 57 | M | no | NA | NA | SAT |
| 44 | 2022 | case series | Spain | 67 | M | no | NA | NA | SAT |
| 44 | 2022 | case series | Spain | 47 | M | no | NA | NA | SAT |
| 44 | 2022 | case series | Spain | 69 | F | no | NA | NA | GD + SAT |
| 15 | 2021 | case report | United Kingdom | middle aged | F | no | no | NA | SAT |
| 16 | 2021 | case series | Italy | 26 | F | no | no | no | SAT |
| 16 | 2021 | case series | Italy | 49 | F | benign thyroid nodule on the paternal side | no | no | SAT |
| 17 | 2021 | letter to the editor | Thailand | 30 | F | father, mother and brother with Grave's disease | no | Graves 'disease since 2018 | Aggravation of GD |
| 18 | 2021 | case series | USA | 37 | M | NA | Prediabetes, dyslipidemia | no | SAT |
| 18 | 2021 | case series | USA | 35 | M | NA | no | no | SAT |
| 18 | 2021 | case series | USA | 41 | F | NA | no | no | Thyroiditis |
| 45 | 2022 | case report | Greece | 36 | F | no | ulcerative gastritis, intraocular hypertension, endometriosis | no | SAT |
| 19 | 2021 | case report | Turkey | 38 | F | NA | no | NA | SAT |
| 20 | 2021 | case report | Cyprus | 40 | F | NA | NA | NA | SAT |
| 46 | 2022 | Letter to the editor | India | 47 | f | NA | NA | NA | SAT |
| 21 | 2021 | Case report | USA | 50 | F | NA | hypertension, anxiety | controlled Graves’ disease without clinical ophthalmopathy since 2010 treated with radioactive iodine consequent hypothyroidism treated with levothyroxine. | TED |
| 47 | 2022 | Case series | Greece | 35 | F | mother with Hashimoto’s thyroiditis, sister SAT at the same time after vaccine | no | no | SAT |
| 47 | 2022 | case series | Greece | 32 | F | mother with Hashimoto’s thyroiditis, sister SAT at the same time after vaccine | endometritis infection 1 month earlier, which was treated with doxycycline and roxithromycin | no | SAT |
| 22 | 2021 | Case series | Turkey | 35 | F | no | no | no | SAT |
| 22 | 2021 | Case series | Turkey | 34 | F | no | mild COVID-19 infection in August 2020 (vaccine 01.2021) | no | SAT |
| 22 | 2021 | Case series | Turkey | 37 | F | no | no | no | SAT |
| 48 | 2022 | Case series | Japan | 38 | F | NA | NA | NA | PT |
| 48 | 2022 | Case series | Japan | 59 | F | NA | NA | NA | PT |
| 23 | 2021 | Case series | USA | 38 | F | no | no | no | GD |
| 23 | 2021 | Case series | USA | 30 | F | sister and aunt with Systemic Erythematosus Lupus | no | no | GD |
| 23 | 2021 | Case series | USA | 63 | M | mother with Graves' disease | no | no | GD |
| 24 | 2021 | case report | Italy | 61 | F | unknow thyroid disordes in 2nd degree relatives | smoking 15 cig/d | no | Hypothyroidism and myxoedema |
| 25 | 2021 | case report | Iran | 34 | F | NA | no | no | SAT |
| 49 | 2022 | case report | USA | 32 | M | no | no | no | GD |
| 50 | 2022 | case report | USA | 48 | M | NA | NA | NA | SAT |
| 26 | 2021 | Case series | Greece | 51 | F | no | no | no | SAT |
| 26 | 2021 | Case series | Greece | 39 | F | mother hypothyroidism Hashimoto | no | no | SAT |
| 27 | 2021 | case report | Belgium | 34 | F | NA | NA | Grave's disease since 2010, remission in 2014 | Recurrence of GD |
| 51 | 2022 | letter to editor | Austria | 71 | F | NA | no | subtotal resection of the left thyroid lobe (reason NA), Graves’ disease treated by thyreostatic drugs until 2004. | Recurrence of GD |
| 51 | 2022 | letter to editor | Austria | 46 | M | NA | NA | no | GD |
| 28 | 2021 | case report | Japan | 64 | F | NA | colorectal cancer, diabetes mellitus, obesity | no | GD |
| 29 | 2021 | case report | Turkey | 67 | m | NA | hypertension | no | SAT |
| 30 | 2021 | case report | Norway | 30 | F | NA | no | Papillary carcinoma and Hashimoto's thyroiditis | SAT |
| 31 | 2021 | case report | USA | 71 | F | NA | stage 4 breast cancer in remission | Struma ovarii at age 35 years, clinically stable multinodular goitre | GD |
| 32 | 2021 | Case series | Turkey | 41 | M | No | no | No | SAT |
| 32 | 2021 | Case series | Turkey | 40 | F | NA | NA | No | SAT |
| 32 | 2021 | Case series | Turkey | 40 | M | No | Smoking for years | No | SAT |
| 32 | 2021 | Case series | Turkey | 26 | F | No | no | No | SAT |
| 32 | 2021 | Case series | Turkey | 44 | F | NA | no | Hashimoto’s thyroiditis | SAT |
| 33 | 2021 | case report | USA | 42 | F | No | stage IIIB pT3N1aM0 right adenocarcinoma of colon (status after right hemicolectomy on 01/2020), followed by adjuvant chemotherapy, paroxysmal supraventricular tachycardia, iron deficiency anemia, chemotherapy-induced neuropathy and lumbar radiculopathy, drinks alcohol socially | No | SAT |
| 34 | 2021 | case report | United Kingdom | 55 | F | No | well-controlled asthma | No | SAT |
| 35 | 2021 | letter to the editor | Australia | 35 | F | both grandmothers with hyperthyroidism | NA | No | GD |
| 35 | 2021 | letter to the editor | Australia | 35 | F | NA | no | Left hemithyroidectomy for a benign thyroid nodule | SAT |
| 36 | 2021 | case series | Spain | 32 | F | NA | no | No | SAT |
| 36 | 2021 | case series | Spain | 33 | F | NA | no | No | SAT |
| 37 | 2021 | case report | Spain | 53 | F | NA | no | No | SAT |
| 38 | 2021 | case report | Brazil | 32 | F | NA | NA | NA | SAT + bilateral optic neuritis |
| 52 | 2022 | case series | Turkey | 61 | M | NA | diabetes mellitus and hypertension for 10 years, s/p Covid in august 2020 | No | SAT |
| 52 | 2022 | case series | Turkey | 32 | F | NA | no | No | SAT |
| 53 | 2022 | case report | South Korea | 34 | M | NA | NA | NA | SAT |
| 54 | 2022 | case series | Turkey | 42 | F | no | NA | Nodular thyroid disease | SAT |
| 54 | 2022 | case series | Turkey | 48 | F | no | Systemic Erythematosus Lupus Eritematosus Systemicus | SAT one month before vaccination | SAT |
| 54 | 2022 | case series | Turkey | 47 | F | no | no autoimmunity, no other information | no | SAT |
| 54 | 2022 | case series | Turkey | 72 | F | no | no autoimmunity, no other information | SAT | SAT |
| 54 | 2022 | case series | Turkey | 50 | M | no | no autoimmunity, no other information | MNG, prior SAT | SAT |
| 54 | 2022 | case series | Turkey | 61 | F | no | no autoimmunity, no other information | MNG | SAT |
| 54 | 2022 | case series | Turkey | 36 | F | no | no autoimmunity, no other information | no | SAT |
| 54 | 2022 | case series | Turkey | 38 | F | no | no autoimmunity, no other information | no | SAT |
| 54 | 2022 | case series | Turkey | 38 | F | no | no autoimmunity, no other information | no | SAT |
| 54 | 2022 | case series | Turkey | 38 | F | family history of LES and mother with Sjogren | no autoimmunity, no other information | no | SAT |
| 54 | 2022 | case series | Turkey | 43 | F | no | no autoimmunity, no other information | no | SAT |
| 54 | 2022 | case series | Turkey | 60 | F | no | personal history of undifferentiated connective tissue disease | MNG, history of subtotal thyroidectomy | SAT |
| 54 | 2022 | case series | Turkey | 46 | F | no | no autoimmunity, no other information | no | SAT |
| 54 | 2022 | case series | Turkey | 34 | F | no | no autoimmunity, no other information | no | SAT |
| 54 | 2022 | case series | Turkey | 71 | M | no | no autoimmunity, no other information | no | SAT |
| 54 | 2022 | case series | Turkey | 40 | F | no | no autoimmunity, no other information | no | GD |
| 54 | 2022 | case series | Turkey | 29 | M | no | no autoimmunity, no other information | no | GD |
| 54 | 2022 | case series | Turkey | 43 | F | no | ankylosing spondilitis | nodular goiter | GD |
| 54 | 2022 | case series | Turkey | 43 | F | no | diabetes insipidus | Hashimoto's thyroiditis | GD |
| 39 | 2021 | letter to editor | South Korea | 46 | F | NA | NA | NA | GD |
| 39 | 2021 | letter to editor | South Korea | 73 | F | NA | NA | NA | GD |
| 39 | 2021 | letter to editor | South Korea | 34 | M | NA | NA | NA | Recurrence of GD |
| 39 | 2021 | letter to editor | South Korea | 39 | F | NA | NA | NA | SAT |
| 39 | 2021 | letter to editor | South Korea | 73 | F | NA | NA | NA | SAT |
| 39 | 2021 | letter to editor | South Korea | 39 | M | NA | NA | NA | GD + SAT |
| 39 | 2021 | letter to editor | South Korea | 33 | M | NA | NA | NA | PT + thyreotoxic periodic paralysis |
| 55 | 2022 | case series | Italy | 32 | M | no | past smoker | no | GD |
| 55 | 2022 | case series | Italy | 35 | M | no | past smoker | no | GD |
| 40 | 2021 | case report | Italy | 52 | M | NA | vitiligo vulgaris, type 2 diabetes | no | GD |
| 56 | 2021 | case report | USA | 42 | F | NA | NA | NA | SAT |
| 42 | 2021 | case report | USA | 57 | F | NA | no | no | SAT |
| 41 | 2021 | case series | Mexico | 40 | F | no | infertility, hypertension | no | GD |
| 41 | 2021 | case series | Mexico | 28 | F | no | NA | no | GD |

| Ref. | Year | Article type | Contry | Age | Gender | Family History | Co-morbidity | Pre-existing thyroid disease | Diagnosis |
| --- | --- | --- | --- | --- | --- | --- | --- | --- | --- |
| 12 | 2021 | case report | China | 40 | F | no | NA | subclinical hypothyroidism (TPO-) | GD |
| 57 | 2021 | case series | Spain | 38 | F | NA | NA | NA | SAT |
| 57 | 2021 | case series | Spain | 32 | M | NA | Type 1 diabetes | NA | PT |
| 57 | 2021 | case series | Spain | 38 | F | NA | Schizophrenia | no | GD |
| 13 | 2021 | case report | Ireland | 42 | F | maternal hypothyroidism | NA | NA | SAT |
| 43 | 2022 | case series | Italy | 29 | F | no | no | no | PT |
| 43 | 2022 | case series | Italy | 34 | M | no | no | no | PT |
| 44 | 2022 | case series | Spain | 71 | F | no | NA | NA | GD |
| 44 | 2022 | case series | Spain | 42 | F | no | NA | NA | GD |
| 44 | 2022 | case series | Spain | 54 | F | no | NA | NA | GD |
| 44 | 2022 | case series | Spain | 46 | F | no | NA | NA | GD |
| 44 | 2022 | case series | Spain | 57 | M | no | NA | NA | SAT |
| 44 | 2022 | case series | Spain | 67 | M | no | NA | NA | SAT |
| 44 | 2022 | case series | Spain | 47 | M | no | NA | NA | SAT |
| 44 | 2022 | case series | Spain | 69 | F | no | NA | NA | GD + SAT |
| 14 | 2021 | case report | United Kingdom | middle aged | F | no | no | NA | SAT |
| 15 | 2021 | case series | Italy | 26 | F | no | no | no | SAT |
| 15 | 2021 | case series | Italy | 49 | F | benign thyroid nodule on the paternal side | no | no | SAT |
| 16 | 2021 | letter to the editor | Thailand | 30 | F | father, mother and brother with Grave's disease | no | Graves 'disease since 2018 | Aggravation of GD |
| 17 | 2021 | case series | USA | 37 | M | NA | Prediabetes, dyslipidemia | no | SAT |
| 17 | 2021 | case series | USA | 35 | M | NA | no | no | SAT |
| 17 | 2021 | case series | USA | 41 | F | NA | no | no | Thyroiditis |
| 45 | 2022 | case report | Greece | 36 | F | no | ulcerative gastritis, intraocular hypertension, endometriosis | no | SAT |
| 18 | 2021 | case report | Turkey | 38 | F | NA | no | NA | SAT |
| 19 | 2021 | case report | Cyprus | 40 | F | NA | NA | NA | SAT |
| 46 | 2022 | Letter to the editor | India | 47 | f | NA | NA | NA | SAT |
| 20 | 2021 | Case report | USA | 50 | F | NA | hypertension, anxiety | controlled Graves’ disease without clinical ophthalmopathy since 2010 treated with radioactive iodine consequent hypothyroidism treated with levothyroxine. | TED |
| 47 | 2022 | Case series | Greece | 35 | F | mother with Hashimoto’s thyroiditis, sister SAT at the same time after vaccine | no | no | SAT |
| 47 | 2022 | case series | Greece | 32 | F | mother with Hashimoto’s thyroiditis, sister SAT at the same time after vaccine | endometritis infection 1 month earlier, which was treated with doxycycline and roxithromycin | no | SAT |
| 21 | 2021 | Case series | Turkey | 35 | F | no | no | no | SAT |
| 21 | 2021 | Case series | Turkey | 34 | F | no | mild COVID-19 infection in August 2020 (vaccine 01.2021) | no | SAT |
| 21 | 2021 | Case series | Turkey | 37 | F | no | no | no | SAT |
| 48 | 2022 | Case series | Japan | 38 | F | NA | NA | NA | PT |
| 48 | 2022 | Case series | Japan | 59 | F | NA | NA | NA | PT |
| 22 | 2021 | Case series | USA | 38 | F | no | no | no | GD |
| 22 | 2021 | Case series | USA | 30 | F | sister and aunt with Systemic Erythematosus Lupus | no | no | GD |
| 22 | 2021 | Case series | USA | 63 | M | mother with Graves' disease | no | no | GD |
| 23 | 2021 | case report | Italy | 61 | F | unknow thyroid disordes in 2nd degree relatives | smoking 15 cig/d | no | Hypothyroidism and myxoedema |
| 24 | 2021 | case report | Iran | 34 | F | NA | no | no | SAT |
| 49 | 2022 | case report | USA | 32 | M | no | no | no | GD |
| 50 | 2022 | case report | USA | 48 | M | NA | NA | NA | SAT |
| 25 | 2021 | Case series | Greece | 51 | F | no | no | no | SAT |
| 25 | 2021 | Case series | Greece | 39 | F | mother hypothyroidism Hashimoto | no | no | SAT |
| 27 | 2021 | case report | Belgium | 34 | F | NA | NA | Grave's disease since 2010, remission in 2014 | Recurrence of GD |
| 51 | 2022 | letter to editor | Austria | 71 | F | NA | no | subtotal resection of the left thyroid lobe (reason NA), Graves’ disease treated by thyreostatic drugs until 2004. | Recurrence of GD |
| 51 | 2022 | letter to editor | Austria | 46 | M | NA | NA | no | GD |
| 28 | 2021 | case report | Japan | 64 | F | NA | colorectal cancer, diabetes mellitus, obesity | no | GD |
| 29 | 2021 | case report | Turkey | 67 | m | NA | hypertension | no | SAT |
| 30 | 2021 | case report | Norway | 30 | F | NA | no | Papillary carcinoma and Hashimoto's thyroiditis | SAT |
| 31 | 2021 | case report | USA | 71 | F | NA | stage 4 breast cancer in remission | Struma ovarii at age 35 years, clinically stable multinodular goitre | GD |
| 32 | 2021 | Case series | Turkey | 41 | M | No | no | No | SAT |
| 32 | 2021 | Case series | Turkey | 40 | F | NA | NA | No | SAT |
| 32 | 2021 | Case series | Turkey | 40 | M | No | Smoking for years | No | SAT |
| 32 | 2021 | Case series | Turkey | 26 | F | No | no | No | SAT |
| 32 | 2021 | Case series | Turkey | 44 | F | NA | no | Hashimoto’s thyroiditis | SAT |
| 33 | 2021 | case report | USA | 42 | F | No | stage IIIB pT3N1aM0 right adenocarcinoma of colon (status after right hemicolectomy on 01/2020), followed by adjuvant chemotherapy, paroxysmal supraventricular tachycardia, iron deficiency anemia, chemotherapy-induced neuropathy and lumbar radiculopathy, drinks alcohol socially | No | SAT |
| 34 | 2021 | case report | United Kingdom | 55 | F | No | well-controlled asthma | No | SAT |
| 35 | 2021 | letter to the editor | Australia | 35 | F | both grandmothers with hyperthyroidism | NA | No | GD |
| 35 | 2021 | letter to the editor | Australia | 35 | F | NA | no | Left hemithyroidectomy for a benign thyroid nodule | SAT |
| 36 | 2021 | case series | Spain | 32 | F | NA | no | No | SAT |
| 36 | 2021 | case series | Spain | 33 | F | NA | no | No | SAT |
| 37 | 2021 | case report | Spain | 53 | F | NA | no | No | SAT |
| 38 | 2021 | case report | Brazil | 32 | F | NA | NA | NA | SAT + bilateral optic neuritis |
| 52 | 2022 | case series | Turkey | 61 | M | NA | diabetes mellitus and hypertension for 10 years, s/p Covid in august 2020 | No | SAT |
| 52 | 2022 | case series | Turkey | 32 | F | NA | no | No | SAT |
| 53 | 2022 | case report | South Korea | 34 | M | NA | NA | NA | SAT |
| 54 | 2022 | case series | Turkey | 42 | F | no | NA | Nodular thyroid disease | SAT |
| 54 | 2022 | case series | Turkey | 48 | F | no | Systemic Erythematosus Lupus Eritematosus Systemicus | SAT one month before vaccination | SAT |
| 54 | 2022 | case series | Turkey | 47 | F | no | no autoimmunity, no other information | no | SAT |
| 54 | 2022 | case series | Turkey | 72 | F | no | no autoimmunity, no other information | SAT | SAT |
| 54 | 2022 | case series | Turkey | 50 | M | no | no autoimmunity, no other information | MNG, prior SAT | SAT |
| 54 | 2022 | case series | Turkey | 61 | F | no | no autoimmunity, no other information | MNG | SAT |
| 54 | 2022 | case series | Turkey | 36 | F | no | no autoimmunity, no other information | no | SAT |
| 54 | 2022 | case series | Turkey | 38 | F | no | no autoimmunity, no other information | no | SAT |
| 54 | 2022 | case series | Turkey | 38 | F | no | no autoimmunity, no other information | no | SAT |
| 54 | 2022 | case series | Turkey | 38 | F | family history of LES and mother with Sjogren | no autoimmunity, no other information | no | SAT |
| 54 | 2022 | case series | Turkey | 43 | F | no | no autoimmunity, no other information | no | SAT |
| 54 | 2022 | case series | Turkey | 60 | F | no | personal history of undifferentiated connective tissue disease | MNG, history of subtotal thyroidectomy | SAT |
| 54 | 2022 | case series | Turkey | 46 | F | no | no autoimmunity, no other information | no | SAT |
| 54 | 2022 | case series | Turkey | 34 | F | no | no autoimmunity, no other information | no | SAT |
| 54 | 2022 | case series | Turkey | 71 | M | no | no autoimmunity, no other information | no | SAT |
| 54 | 2022 | case series | Turkey | 40 | F | no | no autoimmunity, no other information | no | GD |
| 54 | 2022 | case series | Turkey | 29 | M | no | no autoimmunity, no other information | no | GD |
| 54 | 2022 | case series | Turkey | 43 | F | no | ankylosing spondilitis | nodular goiter | GD |
| 54 | 2022 | case series | Turkey | 43 | F | no | diabetes insipidus | Hashimoto's thyroiditis | GD |
| 39 | 2021 | letter to editor | South Korea | 46 | F | NA | NA | NA | GD |
| 39 | 2021 | letter to editor | South Korea | 73 | F | NA | NA | NA | GD |
| 39 | 2021 | letter to editor | South Korea | 34 | M | NA | NA | NA | Recurrence of GD |
| 39 | 2021 | letter to editor | South Korea | 39 | F | NA | NA | NA | SAT |
| 39 | 2021 | letter to editor | South Korea | 73 | F | NA | NA | NA | SAT |
| 39 | 2021 | letter to editor | South Korea | 39 | M | NA | NA | NA | GD + SAT |
| 39 | 2021 | letter to editor | South Korea | 33 | M | NA | NA | NA | PT + thyreotoxic periodic paralysis |
| 55 | 2022 | case series | Italy | 32 | M | no | past smoker | no | GD |
| 55 | 2022 | case series | Italy | 35 | M | no | past smoker | no | GD |
| 40 | 2021 | case report | Italy | 52 | M | NA | vitiligo vulgaris, type 2 diabetes | no | GD |
| 56 | 2021 | case report | USA | 42 | F | NA | NA | NA | SAT |
| 42 | 2021 | case report | USA | 57 | F | NA | no | no | SAT |
| 41 | 2021 | case series | Mexico | 40 | F | no | infertility, hypertension | no | GD |
| 41 | 2021 | case series | Mexico | 28 | F | no | NA | no | GD |

Legend: NA: non available; F: female; M: male; Ref: reference of publication; age expressed in years; SAT: Subacute thyroiditis; GD: Graves' disease; PT: painless thyroiditis
