# Supplementary material for: Thyroid Inconveniences With Vaccination Against SARS-CoV-2: The Size of the Matter. A Systematic Review
Source: Front Endocrinol (Lausanne). 2022 Jun 23;13:900964. doi: 10.3389/fendo.2022.900964 (PMC9259875; doi:10.3389/fendo.2022.900964)
Supplement: Supplementary file 1 [file DataSheet_1.zip › Supplementary Table 2.DOCX]

| **Supplemental Table 2. Symptoms and results of evaluation** | | | | | | |
| --- | --- | --- | --- | --- | --- | --- |
| Ref. | Diagnosis | Symptoms | Physical examination | Thyroid tests | Thyroid Ultrasound | Thyroid scintigraphy |
| 13 | GD | Palpitations | Goiter, thyroid bruit, tachycardia | TSH<0.02 mIU/L (RR 0.47-4.68), fT4 66.6 pmol/L (RR 10.0-28.2), TSI 420% (RR <140%), anti-TPO Ab 239.2 kIU/L (RR <5.6), anti-Tg Ab 7.2 kIU/L (RR <4.1) | heterogeneus background thyroid echogenicity with increase in vascularity | increased uptake |
| 57 | SAT | Cervical pain, tremor, palpitation | Goiter | TSH<0.008 μUIU/mL (RR 0.350-4.950), fT4 1.86 ng/dl (0.70-1.48), anti-Tg Ab 7.40 IU/ml (RR 0-4.11), TPO, TSI Ab negative | enlarged right lobe, diffuse hypoechogenicity | low uptake |
| 57 | PT | Palpitations, insomnia | NA | TSH 0.01 μUIU/mL (RR 0.350-4.950), fT4 2.37 ng/dl (RR 0.70-1.48), TSI Ab 0.4 UI/L (RR, 0.7 UI/L), anti-Tg Ab 42 IU/ml (RR0-4.11), anti-TPO Ab 186 IU/ml (RR 0-5.6) | parenchymal changes compatible with an inflammatory process | low uptake |
| 57 | GD | Nervousness, insomnia, sweating | Goiter | TSH<0.008 μUI/mL (RR 0.350-4.950), fT4 2.01 ng/dl (0.70-1.48), anti-Tg Ab 36.57 IU/ml (RR 0-5.60), anti-TPO Ab 3303.71 IU/mL (RR 0-5.60), TSI Ab 12.54 UI/ml (RR <0.7) | diffuse decrease in echogrnicity with some echogenic septum and increased vascularity | increased uptake |
| 14 | SAT | Cervical pain, fever, palpitations | Goiter, tachycardia | TSH < 0.005 mIU/L (RR 0.27–4.2), fT4 35.3 pmol/L (RR 12–22), CRP 91 mmol/L (RR <5) ESR 60 mm/h (RR<20) anti-TPO Ab 10 IU/mL (RR 0–34), TRAb <1.10 IU/L (RR <1.75) | diffusely heterogeneous, multiple hypoechoic nodules with peripheral and internal vascularity | low uptake |
| 43 | PT | Palpitations, weight loss | Normal | TSH 0.03 μUI/mL (RR 0.2–4.2), fT4 21.7 pmol/L (RR 12–22), anti-Tg Ab negative, anti-TPO Ab negative, TRAb negative | mild hypoechogenicity, difuse heterogeneous echotexture, decreased color Doppler blood flow, small thyroid nodule in the left lobe | low uptake |
| 43 | PT | Palpitations, weight loss | Normal | TSH 0.08 μUI/mL (RR 0.2 –4.2), fT4 15.4 pmol/L (RR 12–22), anti-Tg Ab negative, anti-TPO Ab negative, TRAb negative | hypoechogenicity, diffuse heterogeneous echotexture, decreased color Doppler blood flow signals | low uptake |
| 44 | GD | Weight loss, asthenia | Atrial fibrillation | TSH <0.005 mUI/L (RR 0.38–5.33), fT4 2.3 ng/dl (RR 0.54–1.24), Anti-TPO Ab 30 UI/ml (RR< 9) Anti-Tg Ab ,0.9 UI/ml (RR< 4), TRAb 3.6 U/L (RR< 1.75, ESR 6 mm/h, CRP 5 mg/dl (RR < 10) | Enlarged thyroid, increased vascularity | increased uptake |
| 44 | GD | Weight loss, asthenia, palpitations | NA | TSH <0.005 mUI/L (RR 0.38–5.33), fT4 2.9 ng/dl (RR 0.54–1.24), Anti-TPO Ab 2.5 UI/ml (RR< 9), Anti-TgAb NA, TRAb 4.39 U/L (RR< 1.75), ESR 8 mm/h, CRP 2.5 mg/dl (RR < 10) | Enlarged thyroid, increased vascularity | increased uptake |
| 44 | GD | Weight loss, asthenia, palpitations | NA | TSH <0.005 mUI/L (RR 0.38–5.33), fT4 4.7 ng/dl (RR 0.54–1.24), Anti-TPOAb 30 UI/ml (RR< 9), Anti-TgAb 55 UI/ml (RR< 4), TRAb 5.1 U/L (RR< 1.75) ESR 8 mm/h, CRP 10 mg/dl (RR < 10) | Enlarged thyroid, increased vascularity | NA |
| 44 | GD | Weight loss, palpitation, irritability | NA | TSH <0.005 mUI/L (RR 0.38–5.33), fT4 3.2 ng/dl (RR 0.54–1.24), Anti-TPOAb 60 UI/ml (RR< 9), Anti-Tg Ab 90 UI/ml (RR< 4), TRAb 3.2 U/L (RR< 1.75), ESR 7 mm/h (RR < 10) | Enlarged thyroid, increased vascularity | NA |
| 44 | SAT | Fever, asthenia, weight loss, palpitations | NA | TSH <0.005 mUI/L (RR 0.38–5.33), fT4 5 ng/dl (RR 0.54–1.24), Anti-TPO Ab 7.9 UI/ml (RR< 9), Anti-Tg Ab <0.9 UI/ml (RR< 4), TRAb 0.8 U/L (RR< 1.75), ESR 30 88 mm/h, CRP 88 mg/dl (RR < 10) | Heterogeneous echogenicity, difuse hypoechoic areas, decreased vascularity | NA |
| 44 | SAT | Cervical pain, asthenia, fever | Tachycardia | TSH <0.005 mUI/L (RR 0.38–5.33), fT4 3.5 ng/dl (RR 0.54–1.24), Anti-TPO Ab 10 UI/ml (RR< 9), Anti-TgAb < 0.9UI/ml (RR< 4), TRAb 0.8 U/L (RR< 1.75), ESR 60 mm/h, CRP 120 mg/dl (RR < 10) | Unstructured thyroid, diffuse hypoechoic areas, decreased vascularity | Decreased uptake |
| 44 | SAT | Cervical pain, asthenia, fever | Tachycardia | TSH <0.005 mUI/L (RR 0.38–5.33), fT4 2.6 ng/dl (RR 0.54–1.24), Anti-TPO Ab 0.5 UI/ml (RR< 9), Anti-Tg Ab <0.9 UI/ml (RR< 4), TRAb 0.7 U/L (RR< 1.75), ESR 70 mm/h, CRP 92 mg/dl (RR < 10) | Unstructured thyroid, diffuse hypoechoic areas, decrease vascularity | Decreased uptake |
| 44 | GD + SAT | Cervical pain, fever, weight loss, palpitations, distal tremor | NA | TSH <0.005 mUI/L (RR 0.38–5.33), fT4 1.8 ng/dl (RR 0.54–1.24), Anti-TPO Ab 0.5 UI/ml (RR< 9), Anti-TgAb <0.9 UI/ml (RR< 4), TRAb 3.8 U/L (RR< 1.75), ESR 75 mm/h, CRP 120 mg/dl (RR < 10) | NA | NA |
| 15 | SAT | Cervical pain, insomnia, sweating, hyper-defaecation, weight loss | Goiter | TSH <0.010 mU/L (RR 0.2–4.5), fT4 27 pmol/L (RR 9–21), Anti-TPO Ab 79.5 IU/ml (RR 0–100), TRAb <1.2 IU/L (RR 0-2-1), CRP 23 mg/L (RR < 5) | NA | low uptake |
| 16 | SAT | Cervical pain, fever, chills | Thyroid tenderness | TSH 1.75 mIU/l (RR 0.35–4.94), fT4 9.3 ng/L (RR 7.0–14.8), Anti-TPO Ab negative, Anti-Tg Ab negative, TRAb negative, CRP 29.4 mg/l (RR <5 ) | Distinct ill-defined hypoechoic areas with decreased blood flow | NA |
| 16 | SAT | Cervical pain, headaches | Thyroid tenderness | TSH 0.5 mIU/l (RR 0.35–4.94), fT4 9.4 ng/L (RR 7.0–14.8), Anti-TPO Ab negative, Anti-Tg Ab negative, TRAb negative, CRP 21.9 mg/l (RR <5 ) | NA | NA |
| 17 | Aggravation of GD | Palpitations, weight loss, increased appetite | NA | TSH 0.006 mU/L (RR 0.35-4.94), fT4 1.29 ng/dl (RR 0.7-1.48), TRAb 13.4 IU/L (RR 0-1.75 IU/L) | NA | NA |
| 18 | SAT | Cervical pain, fever | Goiter, tachycardia | TSH < 0.01 mIU/ml (RR: 0.45-4.5), fT4 6.96 ng/dL (RR: 0.82-1.77), TSI Ab negative, anti-TPO Ab negative, Anti-Tg Ab negative, ESR 51 mm/hr (RR 0-10) | heterogeneous, enlarged thyroid gland | decreased uptake |
| 18 | SAT | Cervical pain, palpitations | Tachycardia, thyroid tenderness | TSH < 0.07 mIU/ml (RR: 0.45-4.5), fT4 3.04 ng/dL (RR: 0.82-1.77), TSI Ab negative, anti-TPO Ab negative, Anti-Tg Ab negative | heterogeneous, enlarged thyroid gland | NA |
| 18 | Thyroiditis | Palpitations | Tachycardia | TSH < 0.019 mIU/ml (RR: 0.45-4.5), fT4 2.52 ng/dL (RR: 0.82-1.77), TSI Ab negative, anti-TPO Ab negative, Anti-Tg Ab negative | heterogeneous, enlarged thyroid gland | low uptake |
| 45 | SAT | Cervical pain, fatigue, palpitations | Thyroid tenderness, distal tremor, tachycardia | TSH 0.225 mUI/ml (RR0.4–4), fT4 22.01 pmol/l (RR 12-22), CRP 1.96 mg/dl (RR <0.5), ESR 59 mm/h (RR < 0-20), anti-TPO Ab 15.72 iU/ml (RR < 34), anti-Tg Ab 292 IU/ml (RR <40), TRAb 0.1 U/L (<1.75) | heterogeneous, enlarged thyroid gland with bilateral hypoechoic areas | low uptake |
| 19 | SAT | Cervical pain, fatigue, loss of appetite, sweating | Goiter, thyroid tenderness | TSH 0.008 uIU/mL (RR: 0.27–4.2), fT4 4.65ng/dL (RR: 0.93–1), anti-TPO Ab 9.49 IU/mL (RR: 0–34), anti-Tg Ab 81.58IU/mL (RR: 0–115), PCR 8.76mg/L (RR: 0–0.8), ESR 78mm/h (RR: 0–20) | enlarged right-sided with irregularly demarcated hypoechoic area of approximately 3cm | NA |
| 20 | SAT | Cervical pain, bony aches, exhaustion, emotional lability, palpitations, hyperhidrosis | Thyroid tenderness | TSH 0.11 mIU/L (RR 0.4-4.5), fT4 33.74 pmol/L (RR 10.3-24.46), CRP 174.3 mg/L (RR 0-6), ESR 67 mm/h (RR 0-15), TgAb positive, TRAb and TPOAb negative. | enlarged and heterogeneous thyroid gland with diffusely hypoechoic appearances, reduced vascularity, reactive lymphadenopathy | NA |
| 46 | SAT | Cervical pain, fever, restlessness, weight loss | Goiter, tachycardia, thyroid tenderness | T3 2.2 ng/ml (RR 0.8–2 ng/ml), T4 12.9 µg/dl (RR 4–12 µg/ml), TSH 0.06 µIU/ml (RR 0.2–4.2µIU/ml), antiTPO Ab 11.8 IU/ml (RR <34), TRAb 1.28 IU/ ml (RR< 1.75) | enlarged thyroid gland with hypoechoic nodules | low uptake |
| 21 | TED | Eye irritation, tearing, visual changes, orbital pain | Bilateral proptosis. Clinical activity score: 5. | normal TSH, T4, fT3, TSI 2.29( RR 0–0.55) | NA | NA |
| 47 | SAT | Cervical pain, fatigue, palpitations | Thyroid tenderness | TSH 0.01-0.2 μIU/ml, fT4 increased, Anti-TPO Ab negative, Anti-TG Ab negative, TRAB negative, ESR 75 mm/h (RR <20), CRP 498 mg/L (RR <5) | heterogeneous, enlarged thyroid gland with hypoechogenic regions | low uptake |
| 47 | SAT | Cervical pain, fatigue | Thyroid tenderness | TSH <0.01 μIU/ml, fT4 increased, Anti-TPO Ab negative, Anti-TG Ab negative, TRAB negative, ESR 40 mm/h (RR <20), CRP 10 mg/L (RR <5) | heterogeneous, enlarged thyroid gland with hypoechogenic regions | low uptake |
| 22 | SAT | Cervical pain, fever, fatigue, palpitations | Goiter, thyroid tenderness | TSH 0.473 mIU/L (RR 0.38-5.33), fT4 14.1 pmol/L (RR 7.86-14.41), Anti TPO Ab 1.2 IU/mL (RR 0-9), Anti Tg Ab <0.9 IU/mL (RR 0-4), TRAB <1.5 IU/L (RR <1.5), ESR 53 mm/h (RR<20), CRP 100 mg/L (RR<5) | bilateral focal hypoechoic areas with decreased blood flow on Doppler USG | NA |
| 22 | SAT | Cervical pain, fatigue, fever, palpitation, weight loss | Thyroid tenderness | TSH 0.01 mIU/L (RR 0.38-5.33), fT4 5.2 pmol/L (RR 7.86-14.41), Anti-TPO Ab 1.2 IU/mL (RR 0-9), Anti-Tg Ab <0.9 IU/mL (RR 0-4), TRAB <1.5 IU/L (RR <1.5), ESR 19 mm/h (RR<20), CRP 6 mg/L (RR<5) | Bilateral focal hypoechoic areas with decreased blood flow | NA |
| 22 | SAT | Cervical pain | Thyroid tenderness | TSH 0.9 mIU/L (RR 0.38-5.33), fT4 13.85 pmol/L (RR 7.86-14.41), Anti TPO Ab 4.1 IU/mL (RR 0-9), Anti Tg Ab <0.9 IU/mL (RR 0-4, TRAB <1.5 IU/L (RR <1.5), ESR 25 mm/h (RR<20), CRP 2.4 mg/L (RR<5) | bilateral hypoechoic areas with irregular borders and reduced blood flow in Doppler USG | NA |
| 48 | PT | Palpitations | Goiter | fT4 4.08 ng/dL (RR 0.9–1.7), TSH < 0.005 µIU/mL (RR 0.61–4.23), TgAb 299 IU/mL (0-40), Anti-TPO Ab 350 IU/ml (RR 0-28), TRAb 1.16 IU/L (RR 0-2) | heterogeneous, enlarged thyroid gland, normal Doppler flow | low uptake |
| 48 | PT | Asymptomatic | Normal | TSH 0.01 µIU/mL (RR 0.61-4.23), fT4 2.35 ng/dL(RR 0.9-1.7), TgAb 430 IU/mL (RR 0-40), TPOAb, TRAb negative. | heterogeneous, enlarged thyroid gland, normal blood flow | low uptake |
| 23 | GD | Abdominal pain, gastrointestinal symptoms, fever | Thyroid storm (Burch | TSH <0.008 µIU/mL (RR 0.45-4.5), fT4 108 pmol/L (RR 10.6-22.8), TSI >40 IU/L (RR < 0.5), TRAb 32 IU/L (RR < 1.75), Anti-TPO Ab 1730 IU/ml (RR 0-9) | heterogeneous, enlarged thyroid gland, increased vascularity | NA |
| 23 | GD | Pruritic rash | NA | TSH 0.011 µIU/ml (RR 0.55-4.78), fT4 30.9 pmol/L (RR 11.6-23.2), TRAb 22 IU/L (RR<1.75), Anti-TPO Ab 1149 IU/Ml ( RR 0-9) | heterogeneous, hypervascular thyroid gland with 2 solid isoechoic nodules | elevated uptake |
| 23 | GD | Irritability, palpitations, distal tremor, restless sleep, weight loss | NA | TSH <0.005 µIU/mL (0.45-4.5), fT4 22.9 pmol/L (10.6-22.8), TSI 0.95 IU/L (RR<0.55), Anti-TPO Ab 15 IU/mL (RR 0-34) | NA | NA |
| 24 | Hypothyroidism and myxoedema | Asthenia, weight gain | Goiter, thyroid tenderness, facial and limb swelling | TSH 89.7 mIU/L (RR 0.35–4.50), fT3 Undetectable (RR 2.95–5.41 pmol/L), Anti-TPO Ab >2000 mIU/L (RR <5.6), Anti-Tg Ab 7671 mIU/L (RR <4.1), TRAb 1.2 IU/L (RR <1.75) | heterogeneous,diffuse hypoechoic, enlarged thyroid gland | NA |
| 25 | SAT | Cervical pain, fever, palpitations | Goiter, thyroid tenderness | TSH 0.05 mIU/L (RR 0.36–6.3), T4 20.9 μg/dl (RR 4.4–11.7), ESR 60 (RR 4–9), CRP 9.8 mg/L (RR<10) | heterogeneous thyroid gland with decreased vascularity | low uptake |
| 49 | GD | Palpitations, insomnia, distal tremor, irritability, sweating, dyspnea | Hypertension, tachycardia | TSH <0.005 uIU/mL (RR 0.282–4.000), fT4 5.41 ng/dL (RR 0.84–1.62), TSI 200% (RR <125), Anti-TPO Ab 119 IU/mL (RR<35), Anti-TG Ab 53 IU/mL (RR<40) | heterogeneous thyroid with underlying micronodules suggestive of thyroiditis | 72% homogeneous uptake |
| 50 | SAT | Palpitations, fever, weight loss | Goiter, thyroid tenderness | TSH 0.01 uIU/Ml, elevated CRP and ESR, fT4 3.6 ng/dL | heterogeneous, diffuse hypoechoic, enlarged thyroid gland | NA |
| 26 | SAT | Cervical pain, fever, nausea | Thyroid tenderness | TSH 0.08 mIU/ml (RR 0.38-5.33), fT4 24.84 pmol/L (RR 7.72-17.63), Anti-TPO Ab 0.6 IU/mL (RR 0-9), Anti-Tg Ab <0.9 IU/ml (RR 0-4), TRAb< 0.1 IU/L (RR<1), ESR 103 mm/h (0-30), CRP 135 mg/L (RR<6) | NA | decreased uptake |
| 26 | SAT | Asymptomatic | Normal | TSH <0.03 mIU/ml (RR 0.38-5.33), fT4 20.47 pmol/L (RR 7.72-17.63), Anti-TPO Ab 777.4 IU/mL (RR 0-9), Anti-Tg Ab 257.3 IU/ml (RR 0-4), TRAb < 0.2 IU/L (RR<1), ESR 17 mm/h (0-30), CRP 1 mg/L (RR<6) | diffuse hypoechoic echotexture of the thyroid gland with reduced blood flow | decreased uptake |
| 27 | Recurrence of GD | Distal tremor, sweating, thermophobia, dyspnea, weight loss | Goiter, eyelid oedema, clammy skin, hyperreflexia | TSH <0.01 mU/l (RR 0.4–2.75 mU/l), fT4 2.54 ng/dl (RR 0.75–1.6 ng/dl), TRAb 40 IU/l (RR <0.55 IU/l) | NA | NA |
| 51 | Recurrence of GD | Palpitations, sweating | NA | fT4 3.56 ng/dl (RR 0.70–1.70), TRAb 4.2 IU/l (RR<1.5) | heterogeneous, diffuse hypoechoic thyroid gland with increased vascularization | patchy inhomogenous tracer distribution |
| 51 | GD | NA | NA | fT4 1.63, a fT3 5.18 pg/ml TRAb 2.9 IU/l | heterogeneous, diffuse hypoechoic, enlarged thyroid gland with increased vascularization | patchy, inhomogenous Tc99m accumulation, normal uptake |
| 28 | GD | Palpitations, dyspnea, decreased urine output, leg oedema, fever | Hypertension, atrial fibrillation | TSH < 0.008 mIU/mL, fT3 23.2 ng/dL, fT4 3.32 ng/dL, TRAb positive | enlarged thyroid gland with increased vascularization | NA |
| 29 | SAT | Cervical pain, fever, weight loss | Thyroid palpation, hypertension | TSH 0.005 uIU/ml (RR 0.27–4.2), fT4 1.16 ng/dl (RR 0.93–1.7), ESR 67 mm/h (RR 0–10), CRP 53.9 mg/l (RR 0–5 2.2) | heterogeneous,diffuse hypoechoic thyroid gland | NA |
| 30 | SAT | NA | NA | TSH 3.2–4.7 mIU/L (RR 0.35–3.6), fT4 9.5–13 pmol/L (RR 9–19), Anti-TPO Ab 623 kIU/L (RR < 35) | characteristic of thyroiditis and a hypoechoic nodule in the left lobe | NA |
| 31 | GD | Palpitations, fever, sweating, dyspnea, dizziness, nausea, diarrhoea, abdominal pain, distal tremor | Goiter, tachycardia, leg oedema | TSH <0.02 µ(IU)/ml (RR 0.35–2.00), fT4 7.2 ng/dL (RR 0.9–1.7), TSI 347% (RR <140%), Anti-TPO Ab 8.9 IU/mL (RR 0–9.0) | stable multinodular disease | NA |
| 32 | SAT | Cervical pain, fatigue, palpitations | Goiter, thyroid tenderness, tachycardia | TSH 0.01 mIU/L (RR 0.27–4.2), fT4 3.18 ng/dL (RR 0.93–1.7), ESR 32 mm/h (RR <20), CRP 124 mg/L (RR 0–5), Anti-Tg Ab, Anti-TPO Ab, TRAb negative | heterogeneous,diffuse hypoechoic thyroid gland with decreased blood flow | NA |
| 32 | SAT | Cervical pain, palpitations, sweating | Thyroid tenderness | TSH 0.18 mIU/L (RR 0.27–4.2), fT4 1.58 ng/dL (RR 0.93–1.7), ESR 80 mm/h (RR<2o), CRP 34 mg/L (RR 0–5), Anti-Tg Ab 160 IU/mL (RR 0–115), Anti-TPO Ab, TRAb negative | heterogeneous,diffuse hypoechoic, thyroid gland with decreased blood flow | NA |
| 32 | SAT | Cervical pain, nervousness, fatigue | Goiter, thyroid tenderness | TSH 1.1 mIU/L (RR 0.27–4.2), fT4 1.55 ng/dL (RR 0.93–1.7), ESR 28 mm/h (RR<20), CRP 15 mg/L (RR 0–5), Anti-Tg Ab, Anti-TPO Ab negative | 1.5–2 cm subcapsular heterogeneous hypoechoic thyroiditis area in the left lobe | NA |
| 32 | SAT | Cervical pain | Thyroid tenderness | TSH 0.01 mIU/L (RR 0.27–4.2), fT4 2.02 ng/dL (RR 0.93–1.7), ESR 34 mm/h (RR <20), CRP 27 mg/L (RR 0–5), Anti-Tg Ab 562 IU/mL (RR 0–115), Anti-TPO Ab 424 IU/mL (0–34), TRAb negative | 2 cm thyroiditis area with irregular border, heterogeneous, hypoechoic and decreased blood flow in the left thyroid lobe | low uptake |
| 32 | SAT | Cervical pain, headache, palpitations, sweating, distal tremor | Goiter, thyroid tenderness | TSH 0.24 mIU/L (RR 0.27–4.2), fT4 1.58 ng/dL (RR 0.93–1.7), ESR 44 mm/h (RR <20), CRP 18 mg/L (RR 0–5), Anti-TPO Ab 362 IU/mL (RR 0–75), Anti-Tg Ab, TRAb negative. | heterogeneous,diffuse hypoechoic thyroid gland with decreased blood flow | NA |
| 33 | SAT | Cervical pain | Goiter, thyroid tenderness | TSH < 0.0005 IU/ml (RR 0.350–4.000), fT4-1.51 ng/dL (RR 0.80–2.00), ESR 81 mm/hour, anti-TPO Ab <10 (<35 IU/ml) | 2 bilateral, heterogeneous and diffuse hypoechoic areas in the thyroid gland | NA |
| 34 | SAT | Cervical pain, headache, generalized aches, palpitations | Goiter, thyroid tenderness | TSH 0.09 mU/L (RR 0.3-4.2), fT4 25.2 pmol/L (RR 12.0-22.0), anti-TPO Ab <10 IU/ml (RR <34), CRP 87 mg/L (RR <5), ESR 51 mm/h (RR 0-18) | heterogeneous, enlarged thyroid gland with reduced vascularisation | NA |
| 35 | GD | Palpitations, hyperphagia, thermophobia, distal tremor | NA | TSH < 0.02 mIU/L (RR 0.5-4.0), fT4 64 pmol/L (RR 10-20), TSI 24 IU/L (RR < 0.55), anti-TPO Ab > 1300 IU/mL, anti-Tg Ab 33 IU/mL (RR < 4.5) | diffusely heterogeneous thyroid gland with increased vascularisation | NA |
| 35 | SAT | Cervical pain | NA | TSH, fT4 normal | new 11-mm thyroid imaging and 4 lesion in the right lobe | NA |
| 36 | SAT | Cervical pain, asthenia, malaise, fever | Thyroid tenderness | TSH < 0,008 mUI/L (RR 0,350-4,940), fT4 1,93 ng/dL (RR 0,7-1,48), fT3 6,33 pg/mL (RR 1,59-3,91) | heterogeneous thyroid gland with increased vascularisation | NA |
| 36 | SAT | Cervical pain, fever, asthenia, palpitations, diarrhea and weight loss | Goiter, thyroid tenderness, tachycardia | TSH < 0,008 mUI/L (RR 0,350-4,940), fT4 3,62 ng/dL (RR 0,7-1,48), PCR 55,4 mg/L (RR 0-5 mg/L), ESR 43 ml/h (RR< 25) | heterogeneous, diffuse hypoechoic, enlarged thyroid gland with increased vascularization | NA |
| 37 | SAT | Cervical pain, malaise, asthenia, arthro-myalgias, fever | Goiter, thyroid tenderness, tachycardia | TSH 0,095 mIU/l (RR 0,5-4,5), fT4 1,22 ng/dl (RR 0,7-1,48), ESR 51 mm/h (RR < 15), PCR 10 mg/l (RR < 1,5), anti-TPO Ab 1,42 IU/ml (RR< 5,61), anti-TG Ab 8,4 IU/ml (RR< 4,11), TRAb 1,0 IU/l (RR< 1,5). | enlarged thyroid gland with diffusely hypoechoic areas, decreased vascularity | NA |
| 38 | SAT + bilateral optic neuritis | Symptoms related to optic neuritis, no specific thyroid symptom | Fundus examination: bilateral disc swelling | TSH 13.2 mUI/L (RR 0.45–4.5), normal fT4, anti-Tg Ab, anti-TPO Ab elevated | NA | NA |
| 52 | SAT | Cervical pain, fever, palpitations, fatigue, sweating, weight loss | Goiter, thyroid tenderness, tachycardia | TSH 0.02 mIU/L (RR 0.27-4.2), fT4 27.8 pmol/L (RR 11.97-21.88), anti-TPO Ab 0.51 IU/mL (RR 0-5.61), anti-TG Ab 1.51 IU/mL (RR 0-4.11), TRAb 0.25 IU/L (RR <1.5), CRP 28.6 mg/L (RR<5), ESR 29 mm/h (RR 0-20) | enlarged thyroid gland with devascularized patchy hypoechoic areas in both lobes, more intensely in the right lobe | NA |
| 52 | SAT | Cervical pain, sweating, arthralgia, fever, palpitations, weight loss | Goiter, thyroid tenderness | TSH <0.01 mIU/L (RR 0.27-4.2), fT4 53.5 pmol/L (RR 11.97-21.88), anti-TPO Ab 2.32 IU/mL (RR 0-5.61), anti-Tg Ab 3.41 IU/mL (RR 0-4.11), CRP 24.09 mg/L (RR<5), ESR 62 mm/h (RR 0-20) | diffuse swelling of both thyroid glands. Patchy hypoechoic areas with a lack of flow color on Doppler US in both thyroid lobes, especially in the right lobe | NA |
| 53 | SAT | Fatigue, headache, sweating, weight loss | Goiter, thyroid tenderness, tachycardia | TSH 0.005 µIU/mL (RR 0.4–4.8), fT4 4.35 ng/dL (RR 0.8–1.71), CRP 6.54 mg/dL (NR, 0–0.3), ESR 79 mm/hr (RR 0–26), anti-Tg Ab 11.10 IU/mL (RR 0–115), TSI 0.1 IU/L (RR 0–1.5 IU/L) | typical SAT findings, heterogeneous, enlarged thyroid gland | NA |
| 54 | SAT | * | NA | TSH < 0.015 mIU/L (RR 0.38-5.33), anti-TPO Ab 0.3 IU/mL (RR 0-9), anti-TG Ab 2 IU/mL (RR 0-4), CRP 4.44 mg/L (RR 0-0.8), ESR 74 mm/h (RR 0-25) | Patchy heterogenous hypoechoic areas in the right lobe | partially suppressed thyroid gland |
| 54 | SAT | * | NA | TSH 0.031 mIU/L (RR 0.38-5.33), anti-TPO Ab 0.5 IU/mL (RR 0-9), anti-TG Ab 183.6 IU/mL (RR 0-4), CRP 5.8 mg/L (RR 0-0.8), ESR 48 mm/h (RR: 0-25) | Patchy heterogenous hypoechoic areas | NA |
| 54 | SAT | * | NA | TSH 0.54 mIU/L (RR 0.38-5.33), fT4 13.42pmol/L (RR 7.86-14.41), Tg 41.1 (RR 1.15-50) anti-TPO Ab 5.8 IU/mL (RR 0-9), anti-TG Ab <0.9 IU/mL (RR 0-4), CRP 4.85 mg/L (RR 0-0.8), ESR 55 mm/h (RR: 0-25) | Patchy heterogenous hypoechoic areas | NA |
| 54 | SAT | * | NA | TSH 2.44 mIU/L (RR 0.38-5.33), fT4 11.8 pmol/L (RR 7.86-14.41), anti-TPO <0.25 IU/mL (RR 0-9), CRP 0.77 mg/L (RR 0-0.8), ESR 10 mm/h (RR: 0-25) | Patchy heterogenous hypoechoic areas in the right lobe | NA |
| 54 | SAT | * | NA | TSH 0.127 mIU/L (RR 0.38-5.33), fT4 11.40 pmol/L (RR 7.86-14.41), anti-TPO Ab 2.9 IU/mL (RR 0-9), anti-Tg Ab 7.9 IU/mL (RR 0-4), TRAb 0.9 IU/mL (RR <1.5), CRP 1.02 mg/L (RR 0-0.8), ESR 41 mm/h (RR: 0-25) | ill-defined heterogenous area in the right lobe 50x25 mm | NA |
| 54 | SAT | * | NA | TSH 4.44 mIU/L (RR 0.38-5.33), fT4 10.99 pmol/L (RR 7.86-14.41), anti-TPO Ab 1.2 IU/mL (RR 0-9), anti-TG Ab < 0.9 IU/mL (RR 0-4), TRAb NA IU/mL (RR <1.5), CRP 1.16 mg/L (RR 0-0.8), ESR 344 mm/h (RR: 0-25) | Patchy heterogenous hypoechoic areas | NA |
| 54 | SAT | * | NA | TSH 0.47 mIU/L (RR 0.38-5.33), fT4 19.11 pmol/L (RR 7.86-14.41), anti-TPO Ab 1.2 IU/mL (RR: 0-9), anti-Tg Ab 10.9 IU/mL (RR 0-4), TRAb <1.5 IU/mL (RR <1.5), CRP 10.5 mg/L (RR 0-0.8), ESR 53 mm/h (RR 0-25) | Patchy heterogenous hypoechoic areas, decreased vascularisation | NA |
| 54 | SAT | * | NA | TSH 0.018 mIU/L (RR 0.38-5.33), fT4 26.10 pmol/L (RR 7.86-14.41), anti-TPO Ab 4.1 IU/mL (RR 0-9), anti-Tg Ab <0.9 IU/mL (RR 0-4), TRAb <1.5 IU/mL (RR <1.5), CRP 0.3 mg/L (RR 0-0.8), ESR 44 mm/h (RR: 0-25) | Patchy heterogenous hypoechoic areas, decreased vascularisation | NA |
| 54 | SAT | * | NA | TSH <0.01 mIU/L (RR 0.38-5.33), fT4 51.48 pmol/L (RR 7.86-14.41), anti-TPO Ab 6.11 IU/mL (RR 0-9), anti-TG Ab <0.9 IU/mL (RR 0-4), TRAb 0.45 IU/mL (RR <1.5), CRP 13.63 mg/L (RR 0-0.8), ESR 55mm/h (RR: 0-25) | Patchy heterogenous hypoechoic areas | NA |
| 54 | SAT | * | NA | TSH 0.032 mIU/L (RR 0.38-5.33), fT4 12.23 pmol/L (RR 7.86-14.41), anti-TPO Ab 1.2 IU/mL (RR 0-9), anti-Tg Ab < 0.9 IU/mL (RR 0-4), CRP 1.9 mg/L (RR 0-0.8), ESR 42 mm/h (RR: 0-25) | Patchy heterogenous hypoechoic areas | NA |
| 54 | SAT | * | NA | TSH 0.01 mIU/L (RR 0.38-5.33), fT4 37.7 pmol/L (RR 7.86-14.41), anti-TPO Ab 28 IU/mL (RR 0-9), anti-Tg Ab 26 IU/mL (RR 0-4), TRAb 0.8 IU/mL (RR <1.5), CRP 42.9 mg/L (RR 0-0.8) | Patchy heterogenous hypoechoic areas, decreased vascularisation | low uptake |
| 54 | SAT | * | NA | TSH 0.6 mIU/L (RR 0.38-5.33), fT4 14.0 pmol/L (RR 7.86-14.41), CRP 5.2 mg/L (RR 0-0.8), ESR 33 mm/h (RR 0-25) | Patchy heterogenous hypoechoic area in the middle part of left lobe | NA |
| 54 | SAT | * | NA | TSH 0.43 mIU/L (RR 0.38-5.33), fT4 14.8 pmol/L (RR 7.86-14.41), anti-TPO Ab 0.7 IU/mL (RR 0-9), anti-Tg Ab <0.9 IU/mL (RR 0-4), CRP 1.70 mg/L (RR 0-0.8), ESR 60 mm/h (RR: 0-25) | Patchy heterogenous hypoechoic areas | NA |
| 54 | SAT | * | NA | TSH 0.03 mIU/L (RR 0.38-5.33), fT4 31.65 pmol/L (RR 7.86-14.41), anti-TPO Ab 1.2 IU/mL (RR 0-9), anti-Tg Ab < 0.9IU/mL (RR 0-4), TRAb 3 IU/mL (RR <1.5), CRP 0.6 mg/L (RR 0-0.8), ESR 18 mm/h (RR 0-25) | At SAT diagnosis : Patchy heterogenous hypoechoic areas, decreased vascularisation. At GD diagnosis : diffuse heterogenous thyroid gland | increased uptake |
| 54 | SAT | * | NA | TSH 0.038 mIU/L (RR 0.38-5.33), fT4 17.27 pmol/L (RR 7.86-14.41), anti-TPO Ab 4.7 IU/mL (RR 0-9), anti-TG Ab <0.9 IU/mL (RR 0-4), TRAb 0.82 IU/mL (RR <1.5), CRP 3.65 mg/L (RR 0-0.8), ESR 67 mm/h (RR: 0-25) | Patchy heterogenous hypoechoic areas, decreased vascularisation | NA |
| 54 | GD | * | NA | TSH < 0.015 mIU/L (RR 0.38-5.33), fT4 27.92 pmol/L (RR 7.86-14.41), anti-TPO Ab 195.7 IU/mL (RR 0-9), anti-Tg Ab 7.1 IU/mL (RR 0-4), TRAb 10.3 IU/mL (RR <1.5), CRP 0.84 mg/L (RR 0-0.8), ESR 11 mm/h (RR: 0-25) | Diffuse hyperplasia, increased vascularisation | increased uptake |
| 54 | GD | * | NA | TSH < 0.015 mIU/L (RR 0.38-5.33), fT4 12.15 pmol/L (RR 7.86-14.41), anti-TPO Ab 0.7 IU/mL (RR 0-9), anti-TG Ab <0.9 IU/mL (RR 0-4), TRAb 0.97 IU/mL (RR <1.5), CRP 0.8 mg/L (RR 0-0.8), ESR 18 mm/h (RR 0-25) | Diffuse hyperplasia, increased vascularisation | increased uptake |
| 54 | GD | * | NA | TSH 0.015 mIU/L (RR 0.38-5.33), fT4 33.1 pmol/L (RR 7.86-14.41), anti-TPO Ab 0.8 IU/mL (RR: 0-9), anti-Tg Ab 1.8 IU/mL (RR 0-4), TRAb 0.25 IU/mL (RR <1.5), CRP 0.6 mg/L (RR 0-0.8), ESR 17 mm/h (RR 0-25) | Diffuse hyperplasia, increased vascularisation | increased uptake |
| 54 | GD | * | NA | TSH 0.01 mIU/L (RR 0.38-5.33), fT4 25.5 pmol/L (RR 7.86-14.41), anti-TPO Ab 196 IU/mL (RR 0-9), anti-Tg Ab 167 IU/mL (RR 0-4), TRAb 1.9 IU/mL (RR <1.5), CRP 0.3 mg/L (RR 0-0.8), ESR 6 mm/h (RR: 0-25) | Diffuse hyperplasia, increased vascularisation | increased uptake |
| 39 | GD | Chest pain, dyspnea | NA | TSH 0.010 (RR 0.55-4.78), fT4 33.92 (RR 11.5-22.7), anti-TPO Ab 77.72 (<34), anti-Tg Ab 137.5 (<115), TRAb 6.42 (<1.75), ESR 5 (<20), CRP 0.05 (<5) | Diffuse hyperplasia, increased vascularisation | increased uptake |
| 39 | GD | Weight loss, dyspnea | NA | TSH <0.08 (RR 0.55-4.78), fT4 73.8 (RR 11.5-22.7), anti-TPOAb 41.03(RR <34), anti-TgAb NA, TRAb 6.3 (RR <1.75) | Increased vascularity | increased uptake |
| 39 | Recurrence of GD | Weight loss, palpitations | NA | TSH <0.008 (RR 0.55-4.78), fT4 26.61 (RR 11.5-22.7), anti-TPOAb NA, anti-TgAb NA, TRAb 4.24 (RR <1.75) | Increased vascularity | NA |
| 39 | SAT | Cervical pain | NA | TSH 0.113 (RR 0.55-4.78), fT4 31.4 (RR 11.5-22.7), anti-TPO Ab <15 (RR<34), TRAb <1.1 (<1.75), ESR 63 (<20), CRP 28.6 (<5) | Ill-defined hypoechoic lesions | low uptake |
| 39 | SAT | Cervical pain, fever | NA | TSH 0.012 (RR 0.55-4.78), fT4 94.73 (RR 11.5-22.7), anti-TPOAb <15 (RR <34), anti-TgAb 39.71 (RR <115), TRAb 1.41 (RR <1.75), ESR 85 (RR <20), CRP 34.65 (RR <5) | Ill-defined hypoechoic lesions | NA |
| 39 | GD + SAT | Cervical pain, fever | NA | TSH <0.012 (RR 0.55-4.78), fT4 36.98 (RR 11.5-22.7), anti-TPOAb <15 (RR <34), anti-TgAb 295.1 (RR <115), TRAb 2.9 (RR <1.75), ESR 74 (RR <20), CRP 36.51 (RR <5) | enlarged thyroid gland with ill-defined hypoechoic lesion in left | low uptake |
| 39 | PT + thyreotoxic periodic paralysis | Leg weekness | NA | TSH 0.012 (RR 0.55-4.78), fT4 37.39 (RR 11.5-22.7), anti-TPO Ab <15 (RR<34), anti-Tg Ab 203.3 (RR <115), TRAb < 1.1 (RR <1.75), ESR 37 (RR <20), CRP 5.16 (RR<5) | heterogeneous echogenicity, decreased vascularity | low uptake |
| 55 | GD | Anxiety, palpitations | Tachycardia | TSH 0.005 uIU/Ml, fT4 2.96 ng/dL (RR 0.6–1.12), TRAbs 7.98 IU/L (RR <2.9) | enlarged thyroid gland with pseudonodules, hypervascularization | NA |
| 55 | GD | Headache, nausea, asthenia, palpitations | Tachycardia, eye redness, superior palpebral retraction | TSH 0.004 uIU/Ml, fT4 4.96 ng/dL (RR 0.6–1.12), TRAbs 3.2 IU/L (RR <2.9) | enlarged, hypervascularisated thyroid gland | NA |
| 40 | GD | Fever, weight loss, asthenia | Goiter | TSH <0.004 mIU/L (RR 0.4–4.00), fT4 5.56 ng/dL (RR 0.7–1.7), TRAb 6.48 IU/L (RR 0–1.49), anti Tg Ab 30 IU/mL (RR 0-30), anti-TPO Ab 21 IU/mL (RR 0-10) | heterogeneous, enlarged thyroid gland with increased vascularization | NA |
| 56 | SAT | Cervical pain, palpitations, sweating | Tachycardia | TSH <0.01, fT4 4.58, TPOAb <28, ESR 62 | NA | NA |
| 42 | SAT | Cervical pain, fever | Goiter, thyroid tenderness, tachycardia | TSH <0.008 lU/mL (RR 0.4–4.2), fT4 1.92 ng/dL (RR 0.8–1.5), TRAb <1.10 U/L (RR 0–1.75), TSI <0.10 U/L (RR 0–0.55), anti-TPO Ab <0.5 U/mL (RR 0–5.6), Anti-Tg Ab 3.4 U/mL (RR 0–4.1) | asymmetrically enlarged, hypervascular heterogeneous right thyroid lobe | NA |
| 41 | GD | Nausea, vomiting, fatigue, insomnia, palpitations, distal tremor | Hyperreflexia, arhythmic heart sounds | TSH <0.001lgUi/mL (RR 0.27–4.4), fT4 3.57 ng/dL (RR 0.93–1.71), Anti-Tg Ab 210 Ui/mL(RR 0–44), Anti-TPO Ab 3405 Ui/mL(RR 0–5.6), TRAb 16.56 Ui/L (RR 0–1.75), TSI 380% (RR <140), CRP 2.2 mg/L (RR <7.44) | enlarged and hypervascularisated thyroid gland | NA |
| 41 | GD | Anxiety, insomnia, palpitations, distal tremor | NA | TSH <0.001 lgUi/mL (RR 0.27–4.4), fT4 1.84 ng/dL (RR 0.93–1.71), Anti-Tg Ab 33 Ui/mL (RR 0–44), Anti-TPO Ab 833 Ui/mL(RR 0–5.6),TRAb 5.85 Ui/L (RR 0–1.75), CRP 1.26 mg/L (RR <7.44) | NA | diffuse toxic goiter, cold nodule in the middle third of the right lobe, high radioactive tracer distribution in the rest of the gland |

| Ref. | Diagnosis | Symptoms | Physical examination | Thyroid tests | Thyroid Ultrasound | Thyroid scintigraphy |
| --- | --- | --- | --- | --- | --- | --- |
| 12 | GD | Palpitations | Goiter, thyroid bruit, tachycardia | TSH<0.02 mIU/L (RR 0.47-4.68), fT4 66.6 pmol/L (RR 10.0-28.2), TSI 420% (RR <140%), anti-TPO Ab 239.2 kIU/L (RR <5.6), anti-Tg Ab 7.2 kIU/L (RR <4.1) | heterogeneus background thyroid echogenicity with increase in vascularity | increased uptake |
| 57 | SAT | Cervical pain, tremor, palpitation | Goiter | TSH<0.008 μUIU/mL (RR 0.350-4.950), fT4 1.86 ng/dl (0.70-1.48), anti-Tg Ab 7.40 IU/ml (RR 0-4.11), TPO, TSI Ab negative | enlarged right lobe, diffuse hypoechogenicity | low uptake |
| 57 | PT | Palpitations, insomnia | NA | TSH 0.01 μUIU/mL (RR 0.350-4.950), fT4 2.37 ng/dl (RR 0.70-1.48), TSI Ab 0.4 UI/L (RR, 0.7 UI/L), anti-Tg Ab 42 IU/ml (RR0-4.11), anti-TPO Ab 186 IU/ml (RR 0-5.6) | parenchymal changes compatible with an inflammatory process | low uptake |
| 57 | GD | Nervousness, insomnia, sweating | Goiter | TSH<0.008 μUI/mL (RR 0.350-4.950), fT4 2.01 ng/dl (0.70-1.48), anti-Tg Ab 36.57 IU/ml (RR 0-5.60), anti-TPO Ab 3303.71 IU/mL (RR 0-5.60), TSI Ab 12.54 UI/ml (RR <0.7) | diffuse decrease in echogrnicity with some echogenic septum and increased vascularity | increased uptake |
| 13 | SAT | Cervical pain, fever, palpitations | Goiter, tachycardia | TSH < 0.005 mIU/L (RR 0.27–4.2), fT4 35.3 pmol/L (RR 12–22), CRP 91 mmol/L (RR <5) ESR 60 mm/h (RR<20) anti-TPO Ab 10 IU/mL (RR 0–34), TRAb <1.10 IU/L (RR <1.75) | diffusely heterogeneous, multiple hypoechoic nodules with peripheral and internal vascularity | low uptake |
| 43 | PT | Palpitations, weight loss | Normal | TSH 0.03 μUI/mL (RR 0.2–4.2), fT4 21.7 pmol/L (RR 12–22), anti-Tg Ab negative, anti-TPO Ab negative, TRAb negative | mild hypoechogenicity, difuse heterogeneous echotexture, decreased color Doppler blood flow, small thyroid nodule in the left lobe | low uptake |
| 43 | PT | Palpitations, weight loss | Normal | TSH 0.08 μUI/mL (RR 0.2 –4.2), fT4 15.4 pmol/L (RR 12–22), anti-Tg Ab negative, anti-TPO Ab negative, TRAb negative | hypoechogenicity, diffuse heterogeneous echotexture, decreased color Doppler blood flow signals | low uptake |
| 44 | GD | Weight loss, asthenia | Atrial fibrillation | TSH <0.005 mUI/L (RR 0.38–5.33), fT4 2.3 ng/dl (RR 0.54–1.24), Anti-TPO Ab 30 UI/ml (RR< 9) Anti-Tg Ab ,0.9 UI/ml (RR< 4), TRAb 3.6 U/L (RR< 1.75, ESR 6 mm/h, CRP 5 mg/dl (RR < 10) | Enlarged thyroid, increased vascularity | increased uptake |
| 44 | GD | Weight loss, asthenia, palpitations | NA | TSH <0.005 mUI/L (RR 0.38–5.33), fT4 2.9 ng/dl (RR 0.54–1.24), Anti-TPO Ab 2.5 UI/ml (RR< 9), Anti-TgAb NA, TRAb 4.39 U/L (RR< 1.75), ESR 8 mm/h, CRP 2.5 mg/dl (RR < 10) | Enlarged thyroid, increased vascularity | increased uptake |
| 44 | GD | Weight loss, asthenia, palpitations | NA | TSH <0.005 mUI/L (RR 0.38–5.33), fT4 4.7 ng/dl (RR 0.54–1.24), Anti-TPOAb 30 UI/ml (RR< 9), Anti-TgAb 55 UI/ml (RR< 4), TRAb 5.1 U/L (RR< 1.75) ESR 8 mm/h, CRP 10 mg/dl (RR < 10) | Enlarged thyroid, increased vascularity | NA |
| 44 | GD | Weight loss, palpitation, irritability | NA | TSH <0.005 mUI/L (RR 0.38–5.33), fT4 3.2 ng/dl (RR 0.54–1.24), Anti-TPOAb 60 UI/ml (RR< 9), Anti-Tg Ab 90 UI/ml (RR< 4), TRAb 3.2 U/L (RR< 1.75), ESR 7 mm/h (RR < 10) | Enlarged thyroid, increased vascularity | NA |
| 44 | SAT | Fever, asthenia, weight loss, palpitations | NA | TSH <0.005 mUI/L (RR 0.38–5.33), fT4 5 ng/dl (RR 0.54–1.24), Anti-TPO Ab 7.9 UI/ml (RR< 9), Anti-Tg Ab <0.9 UI/ml (RR< 4), TRAb 0.8 U/L (RR< 1.75), ESR 30 88 mm/h, CRP 88 mg/dl (RR < 10) | Heterogeneous echogenicity, difuse hypoechoic areas, decreased vascularity | NA |
| 44 | SAT | Cervical pain, asthenia, fever | Tachycardia | TSH <0.005 mUI/L (RR 0.38–5.33), fT4 3.5 ng/dl (RR 0.54–1.24), Anti-TPO Ab 10 UI/ml (RR< 9), Anti-TgAb < 0.9UI/ml (RR< 4), TRAb 0.8 U/L (RR< 1.75), ESR 60 mm/h, CRP 120 mg/dl (RR < 10) | Unstructured thyroid, diffuse hypoechoic areas, decreased vascularity | Decreased uptake |
| 44 | SAT | Cervical pain, asthenia, fever | Tachycardia | TSH <0.005 mUI/L (RR 0.38–5.33), fT4 2.6 ng/dl (RR 0.54–1.24), Anti-TPO Ab 0.5 UI/ml (RR< 9), Anti-Tg Ab <0.9 UI/ml (RR< 4), TRAb 0.7 U/L (RR< 1.75), ESR 70 mm/h, CRP 92 mg/dl (RR < 10) | Unstructured thyroid, diffuse hypoechoic areas, decrease vascularity | Decreased uptake |
| 44 | GD + SAT | Cervical pain, fever, weight loss, palpitations, distal tremor | NA | TSH <0.005 mUI/L (RR 0.38–5.33), fT4 1.8 ng/dl (RR 0.54–1.24), Anti-TPO Ab 0.5 UI/ml (RR< 9), Anti-TgAb <0.9 UI/ml (RR< 4), TRAb 3.8 U/L (RR< 1.75), ESR 75 mm/h, CRP 120 mg/dl (RR < 10) | NA | NA |
| 14 | SAT | Cervical pain, insomnia, sweating, hyper-defaecation, weight loss | Goiter | TSH <0.010 mU/L (RR 0.2–4.5), fT4 27 pmol/L (RR 9–21), Anti-TPO Ab 79.5 IU/ml (RR 0–100), TRAb <1.2 IU/L (RR 0-2-1), CRP 23 mg/L (RR < 5) | NA | low uptake |
| 15 | SAT | Cervical pain, fever, chills | Thyroid tenderness | TSH 1.75 mIU/l (RR 0.35–4.94), fT4 9.3 ng/L (RR 7.0–14.8), Anti-TPO Ab negative, Anti-Tg Ab negative, TRAb negative, CRP 29.4 mg/l (RR <5 ) | Distinct ill-defined hypoechoic areas with decreased blood flow | NA |
| 15 | SAT | Cervical pain, headaches | Thyroid tenderness | TSH 0.5 mIU/l (RR 0.35–4.94), fT4 9.4 ng/L (RR 7.0–14.8), Anti-TPO Ab negative, Anti-Tg Ab negative, TRAb negative, CRP 21.9 mg/l (RR <5 ) | NA | NA |
| 16 | Aggravation of GD | Palpitations, weight loss, increased appetite | NA | TSH 0.006 mU/L (RR 0.35-4.94), fT4 1.29 ng/dl (RR 0.7-1.48), TRAb 13.4 IU/L (RR 0-1.75 IU/L) | NA | NA |
| 17 | SAT | Cervical pain, fever | Goiter, tachycardia | TSH < 0.01 mIU/ml (RR: 0.45-4.5), fT4 6.96 ng/dL (RR: 0.82-1.77), TSI Ab negative, anti-TPO Ab negative, Anti-Tg Ab negative, ESR 51 mm/hr (RR 0-10) | heterogeneous, enlarged thyroid gland | decreased uptake |
| 17 | SAT | Cervical pain, palpitations | Tachycardia, thyroid tenderness | TSH < 0.07 mIU/ml (RR: 0.45-4.5), fT4 3.04 ng/dL (RR: 0.82-1.77), TSI Ab negative, anti-TPO Ab negative, Anti-Tg Ab negative | heterogeneous, enlarged thyroid gland | NA |
| 17 | Thyroiditis | Palpitations | Tachycardia | TSH < 0.019 mIU/ml (RR: 0.45-4.5), fT4 2.52 ng/dL (RR: 0.82-1.77), TSI Ab negative, anti-TPO Ab negative, Anti-Tg Ab negative | heterogeneous, enlarged thyroid gland | low uptake |
| 45 | SAT | Cervical pain, fatigue, palpitations | Thyroid tenderness, distal tremor, tachycardia | TSH 0.225 mUI/ml (RR0.4–4), fT4 22.01 pmol/l (RR 12-22), CRP 1.96 mg/dl (RR <0.5), ESR 59 mm/h (RR < 0-20), anti-TPO Ab 15.72 iU/ml (RR < 34), anti-Tg Ab 292 IU/ml (RR <40), TRAb 0.1 U/L (<1.75) | heterogeneous, enlarged thyroid gland with bilateral hypoechoic areas | low uptake |
| 18 | SAT | Cervical pain, fatigue, loss of appetite, sweating | Goiter, thyroid tenderness | TSH 0.008 uIU/mL (RR: 0.27–4.2), fT4 4.65ng/dL (RR: 0.93–1), anti-TPO Ab 9.49 IU/mL (RR: 0–34), anti-Tg Ab 81.58IU/mL (RR: 0–115), PCR 8.76mg/L (RR: 0–0.8), ESR 78mm/h (RR: 0–20) | enlarged right-sided with irregularly demarcated hypoechoic area of approximately 3cm | NA |
| 19 | SAT | Cervical pain, bony aches, exhaustion, emotional lability, palpitations, hyperhidrosis | Thyroid tenderness | TSH 0.11 mIU/L (RR 0.4-4.5), fT4 33.74 pmol/L (RR 10.3-24.46), CRP 174.3 mg/L (RR 0-6), ESR 67 mm/h (RR 0-15), TgAb positive, TRAb and TPOAb negative. | enlarged and heterogeneous thyroid gland with diffusely hypoechoic appearances, reduced vascularity, reactive lymphadenopathy | NA |
| 46 | SAT | Cervical pain, fever, restlessness, weight loss | Goiter, tachycardia, thyroid tenderness | T3 2.2 ng/ml (RR 0.8–2 ng/ml), T4 12.9 µg/dl (RR 4–12 µg/ml), TSH 0.06 µIU/ml (RR 0.2–4.2µIU/ml), antiTPO Ab 11.8 IU/ml (RR <34), TRAb 1.28 IU/ ml (RR< 1.75) | enlarged thyroid gland with hypoechoic nodules | low uptake |
| 20 | TED | Eye irritation, tearing, visual changes, orbital pain | Bilateral proptosis. Clinical activity score: 5. | normal TSH, T4, fT3, TSI 2.29( RR 0–0.55) | NA | NA |
| 47 | SAT | Cervical pain, fatigue, palpitations | Thyroid tenderness | TSH 0.01-0.2 μIU/ml, fT4 increased, Anti-TPO Ab negative, Anti-TG Ab negative, TRAB negative, ESR 75 mm/h (RR <20), CRP 498 mg/L (RR <5) | heterogeneous, enlarged thyroid gland with hypoechogenic regions | low uptake |
| 47 | SAT | Cervical pain, fatigue | Thyroid tenderness | TSH <0.01 μIU/ml, fT4 increased, Anti-TPO Ab negative, Anti-TG Ab negative, TRAB negative, ESR 40 mm/h (RR <20), CRP 10 mg/L (RR <5) | heterogeneous, enlarged thyroid gland with hypoechogenic regions | low uptake |
| 21 | SAT | Cervical pain, fever, fatigue, palpitations | Goiter, thyroid tenderness | TSH 0.473 mIU/L (RR 0.38-5.33), fT4 14.1 pmol/L (RR 7.86-14.41), Anti TPO Ab 1.2 IU/mL (RR 0-9), Anti Tg Ab <0.9 IU/mL (RR 0-4), TRAB <1.5 IU/L (RR <1.5), ESR 53 mm/h (RR<20), CRP 100 mg/L (RR<5) | bilateral focal hypoechoic areas with decreased blood flow on Doppler USG | NA |
| 21 | SAT | Cervical pain, fatigue, fever, palpitation, weight loss | Thyroid tenderness | TSH 0.01 mIU/L (RR 0.38-5.33), fT4 5.2 pmol/L (RR 7.86-14.41), Anti-TPO Ab 1.2 IU/mL (RR 0-9), Anti-Tg Ab <0.9 IU/mL (RR 0-4), TRAB <1.5 IU/L (RR <1.5), ESR 19 mm/h (RR<20), CRP 6 mg/L (RR<5) | Bilateral focal hypoechoic areas with decreased blood flow | NA |
| 21 | SAT | Cervical pain | Thyroid tenderness | TSH 0.9 mIU/L (RR 0.38-5.33), fT4 13.85 pmol/L (RR 7.86-14.41), Anti TPO Ab 4.1 IU/mL (RR 0-9), Anti Tg Ab <0.9 IU/mL (RR 0-4, TRAB <1.5 IU/L (RR <1.5), ESR 25 mm/h (RR<20), CRP 2.4 mg/L (RR<5) | bilateral hypoechoic areas with irregular borders and reduced blood flow in Doppler USG | NA |
| 48 | PT | Palpitations | Goiter | fT4 4.08 ng/dL (RR 0.9–1.7), TSH < 0.005 µIU/mL (RR 0.61–4.23), TgAb 299 IU/mL (0-40), Anti-TPO Ab 350 IU/ml (RR 0-28), TRAb 1.16 IU/L (RR 0-2) | heterogeneous, enlarged thyroid gland, normal Doppler flow | low uptake |
| 48 | PT | Asymptomatic | Normal | TSH 0.01 µIU/mL (RR 0.61-4.23), fT4 2.35 ng/dL(RR 0.9-1.7), TgAb 430 IU/mL (RR 0-40), TPOAb, TRAb negative. | heterogeneous, enlarged thyroid gland, normal blood flow | low uptake |
| 22 | GD | Abdominal pain, gastrointestinal symptoms, fever | Thyroid storm (Burch | TSH <0.008 µIU/mL (RR 0.45-4.5), fT4 108 pmol/L (RR 10.6-22.8), TSI >40 IU/L (RR < 0.5), TRAb 32 IU/L (RR < 1.75), Anti-TPO Ab 1730 IU/ml (RR 0-9) | heterogeneous, enlarged thyroid gland, increased vascularity | NA |
| 22 | GD | Pruritic rash | NA | TSH 0.011 µIU/ml (RR 0.55-4.78), fT4 30.9 pmol/L (RR 11.6-23.2), TRAb 22 IU/L (RR<1.75), Anti-TPO Ab 1149 IU/Ml ( RR 0-9) | heterogeneous, hypervascular thyroid gland with 2 solid isoechoic nodules | elevated uptake |
| 22 | GD | Irritability, palpitations, distal tremor, restless sleep, weight loss | NA | TSH <0.005 µIU/mL (0.45-4.5), fT4 22.9 pmol/L (10.6-22.8), TSI 0.95 IU/L (RR<0.55), Anti-TPO Ab 15 IU/mL (RR 0-34) | NA | NA |
| 23 | Hypothyroidism and myxoedema | Asthenia, weight gain | Goiter, thyroid tenderness, facial and limb swelling | TSH 89.7 mIU/L (RR 0.35–4.50), fT3 Undetectable (RR 2.95–5.41 pmol/L), Anti-TPO Ab >2000 mIU/L (RR <5.6), Anti-Tg Ab 7671 mIU/L (RR <4.1), TRAb 1.2 IU/L (RR <1.75) | heterogeneous,diffuse hypoechoic, enlarged thyroid gland | NA |
| 24 | SAT | Cervical pain, fever, palpitations | Goiter, thyroid tenderness | TSH 0.05 mIU/L (RR 0.36–6.3), T4 20.9 μg/dl (RR 4.4–11.7), ESR 60 (RR 4–9), CRP 9.8 mg/L (RR<10) | heterogeneous thyroid gland with decreased vascularity | low uptake |
| 49 | GD | Palpitations, insomnia, distal tremor, irritability, sweating, dyspnea | Hypertension, tachycardia | TSH <0.005 uIU/mL (RR 0.282–4.000), fT4 5.41 ng/dL (RR 0.84–1.62), TSI 200% (RR <125), Anti-TPO Ab 119 IU/mL (RR<35), Anti-TG Ab 53 IU/mL (RR<40) | heterogeneous thyroid with underlying micronodules suggestive of thyroiditis | 72% homogeneous uptake |
| 50 | SAT | Palpitations, fever, weight loss | Goiter, thyroid tenderness | TSH 0.01 uIU/Ml, elevated CRP and ESR, fT4 3.6 ng/dL | heterogeneous, diffuse hypoechoic, enlarged thyroid gland | NA |
| 25 | SAT | Cervical pain, fever, nausea | Thyroid tenderness | TSH 0.08 mIU/ml (RR 0.38-5.33), fT4 24.84 pmol/L (RR 7.72-17.63), Anti-TPO Ab 0.6 IU/mL (RR 0-9), Anti-Tg Ab <0.9 IU/ml (RR 0-4), TRAb< 0.1 IU/L (RR<1), ESR 103 mm/h (0-30), CRP 135 mg/L (RR<6) | NA | decreased uptake |
| 25 | SAT | Asymptomatic | Normal | TSH <0.03 mIU/ml (RR 0.38-5.33), fT4 20.47 pmol/L (RR 7.72-17.63), Anti-TPO Ab 777.4 IU/mL (RR 0-9), Anti-Tg Ab 257.3 IU/ml (RR 0-4), TRAb < 0.2 IU/L (RR<1), ESR 17 mm/h (0-30), CRP 1 mg/L (RR<6) | diffuse hypoechoic echotexture of the thyroid gland with reduced blood flow | decreased uptake |
| 27 | Recurrence of GD | Distal tremor, sweating, thermophobia, dyspnea, weight loss | Goiter, eyelid oedema, clammy skin, hyperreflexia | TSH <0.01 mU/l (RR 0.4–2.75 mU/l), fT4 2.54 ng/dl (RR 0.75–1.6 ng/dl), TRAb 40 IU/l (RR <0.55 IU/l) | NA | NA |
| 51 | Recurrence of GD | Palpitations, sweating | NA | fT4 3.56 ng/dl (RR 0.70–1.70), TRAb 4.2 IU/l (RR<1.5) | heterogeneous, diffuse hypoechoic thyroid gland with increased vascularization | patchy inhomogenous tracer distribution |
| 51 | GD | NA | NA | fT4 1.63, a fT3 5.18 pg/ml TRAb 2.9 IU/l | heterogeneous, diffuse hypoechoic, enlarged thyroid gland with increased vascularization | patchy, inhomogenous Tc99m accumulation, normal uptake |
| 28 | GD | Palpitations, dyspnea, decreased urine output, leg oedema, fever | Hypertension, atrial fibrillation | TSH < 0.008 mIU/mL, fT3 23.2 ng/dL, fT4 3.32 ng/dL, TRAb positive | enlarged thyroid gland with increased vascularization | NA |
| 29 | SAT | Cervical pain, fever, weight loss | Thyroid palpation, hypertension | TSH 0.005 uIU/ml (RR 0.27–4.2), fT4 1.16 ng/dl (RR 0.93–1.7), ESR 67 mm/h (RR 0–10), CRP 53.9 mg/l (RR 0–5 2.2) | heterogeneous,diffuse hypoechoic thyroid gland | NA |
| 30 | SAT | NA | NA | TSH 3.2–4.7 mIU/L (RR 0.35–3.6), fT4 9.5–13 pmol/L (RR 9–19), Anti-TPO Ab 623 kIU/L (RR < 35) | characteristic of thyroiditis and a hypoechoic nodule in the left lobe | NA |
| 31 | GD | Palpitations, fever, sweating, dyspnea, dizziness, nausea, diarrhoea, abdominal pain, distal tremor | Goiter, tachycardia, leg oedema | TSH <0.02 µ(IU)/ml (RR 0.35–2.00), fT4 7.2 ng/dL (RR 0.9–1.7), TSI 347% (RR <140%), Anti-TPO Ab 8.9 IU/mL (RR 0–9.0) | stable multinodular disease | NA |
| 32 | SAT | Cervical pain, fatigue, palpitations | Goiter, thyroid tenderness, tachycardia | TSH 0.01 mIU/L (RR 0.27–4.2), fT4 3.18 ng/dL (RR 0.93–1.7), ESR 32 mm/h (RR <20), CRP 124 mg/L (RR 0–5), Anti-Tg Ab, Anti-TPO Ab, TRAb negative | heterogeneous,diffuse hypoechoic thyroid gland with decreased blood flow | NA |
| 32 | SAT | Cervical pain, palpitations, sweating | Thyroid tenderness | TSH 0.18 mIU/L (RR 0.27–4.2), fT4 1.58 ng/dL (RR 0.93–1.7), ESR 80 mm/h (RR<2o), CRP 34 mg/L (RR 0–5), Anti-Tg Ab 160 IU/mL (RR 0–115), Anti-TPO Ab, TRAb negative | heterogeneous,diffuse hypoechoic, thyroid gland with decreased blood flow | NA |
| 32 | SAT | Cervical pain, nervousness, fatigue | Goiter, thyroid tenderness | TSH 1.1 mIU/L (RR 0.27–4.2), fT4 1.55 ng/dL (RR 0.93–1.7), ESR 28 mm/h (RR<20), CRP 15 mg/L (RR 0–5), Anti-Tg Ab, Anti-TPO Ab negative | 1.5–2 cm subcapsular heterogeneous hypoechoic thyroiditis area in the left lobe | NA |
| 32 | SAT | Cervical pain | Thyroid tenderness | TSH 0.01 mIU/L (RR 0.27–4.2), fT4 2.02 ng/dL (RR 0.93–1.7), ESR 34 mm/h (RR <20), CRP 27 mg/L (RR 0–5), Anti-Tg Ab 562 IU/mL (RR 0–115), Anti-TPO Ab 424 IU/mL (0–34), TRAb negative | 2 cm thyroiditis area with irregular border, heterogeneous, hypoechoic and decreased blood flow in the left thyroid lobe | low uptake |
| 32 | SAT | Cervical pain, headache, palpitations, sweating, distal tremor | Goiter, thyroid tenderness | TSH 0.24 mIU/L (RR 0.27–4.2), fT4 1.58 ng/dL (RR 0.93–1.7), ESR 44 mm/h (RR <20), CRP 18 mg/L (RR 0–5), Anti-TPO Ab 362 IU/mL (RR 0–75), Anti-Tg Ab, TRAb negative. | heterogeneous,diffuse hypoechoic thyroid gland with decreased blood flow | NA |
| 33 | SAT | Cervical pain | Goiter, thyroid tenderness | TSH < 0.0005 IU/ml (RR 0.350–4.000), fT4-1.51 ng/dL (RR 0.80–2.00), ESR 81 mm/hour, anti-TPO Ab <10 (<35 IU/ml) | 2 bilateral, heterogeneous and diffuse hypoechoic areas in the thyroid gland | NA |
| 34 | SAT | Cervical pain, headache, generalized aches, palpitations | Goiter, thyroid tenderness | TSH 0.09 mU/L (RR 0.3-4.2), fT4 25.2 pmol/L (RR 12.0-22.0), anti-TPO Ab <10 IU/ml (RR <34), CRP 87 mg/L (RR <5), ESR 51 mm/h (RR 0-18) | heterogeneous, enlarged thyroid gland with reduced vascularisation | NA |
| 35 | GD | Palpitations, hyperphagia, thermophobia, distal tremor | NA | TSH < 0.02 mIU/L (RR 0.5-4.0), fT4 64 pmol/L (RR 10-20), TSI 24 IU/L (RR < 0.55), anti-TPO Ab > 1300 IU/mL, anti-Tg Ab 33 IU/mL (RR < 4.5) | diffusely heterogeneous thyroid gland with increased vascularisation | NA |
| 35 | SAT | Cervical pain | NA | TSH, fT4 normal | new 11-mm thyroid imaging and 4 lesion in the right lobe | NA |
| 36 | SAT | Cervical pain, asthenia, malaise, fever | Thyroid tenderness | TSH < 0,008 mUI/L (RR 0,350-4,940), fT4 1,93 ng/dL (RR 0,7-1,48), fT3 6,33 pg/mL (RR 1,59-3,91) | heterogeneous thyroid gland with increased vascularisation | NA |
| 36 | SAT | Cervical pain, fever, asthenia, palpitations, diarrhea and weight loss | Goiter, thyroid tenderness, tachycardia | TSH < 0,008 mUI/L (RR 0,350-4,940), fT4 3,62 ng/dL (RR 0,7-1,48), PCR 55,4 mg/L (RR 0-5 mg/L), ESR 43 ml/h (RR< 25) | heterogeneous, diffuse hypoechoic, enlarged thyroid gland with increased vascularization | NA |
| 37 | SAT | Cervical pain, malaise, asthenia, arthro-myalgias, fever | Goiter, thyroid tenderness, tachycardia | TSH 0,095 mIU/l (RR 0,5-4,5), fT4 1,22 ng/dl (RR 0,7-1,48), ESR 51 mm/h (RR < 15), PCR 10 mg/l (RR < 1,5), anti-TPO Ab 1,42 IU/ml (RR< 5,61), anti-TG Ab 8,4 IU/ml (RR< 4,11), TRAb 1,0 IU/l (RR< 1,5). | enlarged thyroid gland with diffusely hypoechoic areas, decreased vascularity | NA |
| 38 | SAT + bilateral optic neuritis | Symptoms related to optic neuritis, no specific thyroid symptom | Fundus examination: bilateral disc swelling | TSH 13.2 mUI/L (RR 0.45–4.5), normal fT4, anti-Tg Ab, anti-TPO Ab elevated | NA | NA |
| 52 | SAT | Cervical pain, fever, palpitations, fatigue, sweating, weight loss | Goiter, thyroid tenderness, tachycardia | TSH 0.02 mIU/L (RR 0.27-4.2), fT4 27.8 pmol/L (RR 11.97-21.88), anti-TPO Ab 0.51 IU/mL (RR 0-5.61), anti-TG Ab 1.51 IU/mL (RR 0-4.11), TRAb 0.25 IU/L (RR <1.5), CRP 28.6 mg/L (RR<5), ESR 29 mm/h (RR 0-20) | enlarged thyroid gland with devascularized patchy hypoechoic areas in both lobes, more intensely in the right lobe | NA |
| 52 | SAT | Cervical pain, sweating, arthralgia, fever, palpitations, weight loss | Goiter, thyroid tenderness | TSH <0.01 mIU/L (RR 0.27-4.2), fT4 53.5 pmol/L (RR 11.97-21.88), anti-TPO Ab 2.32 IU/mL (RR 0-5.61), anti-Tg Ab 3.41 IU/mL (RR 0-4.11), CRP 24.09 mg/L (RR<5), ESR 62 mm/h (RR 0-20) | diffuse swelling of both thyroid glands. Patchy hypoechoic areas with a lack of flow color on Doppler US in both thyroid lobes, especially in the right lobe | NA |
| 53 | SAT | Fatigue, headache, sweating, weight loss | Goiter, thyroid tenderness, tachycardia | TSH 0.005 µIU/mL (RR 0.4–4.8), fT4 4.35 ng/dL (RR 0.8–1.71), CRP 6.54 mg/dL (NR, 0–0.3), ESR 79 mm/hr (RR 0–26), anti-Tg Ab 11.10 IU/mL (RR 0–115), TSI 0.1 IU/L (RR 0–1.5 IU/L) | typical SAT findings, heterogeneous, enlarged thyroid gland | NA |
| 54 | SAT | * | NA | TSH < 0.015 mIU/L (RR 0.38-5.33), anti-TPO Ab 0.3 IU/mL (RR 0-9), anti-TG Ab 2 IU/mL (RR 0-4), CRP 4.44 mg/L (RR 0-0.8), ESR 74 mm/h (RR 0-25) | Patchy heterogenous hypoechoic areas in the right lobe | partially suppressed thyroid gland |
| 54 | SAT | * | NA | TSH 0.031 mIU/L (RR 0.38-5.33), anti-TPO Ab 0.5 IU/mL (RR 0-9), anti-TG Ab 183.6 IU/mL (RR 0-4), CRP 5.8 mg/L (RR 0-0.8), ESR 48 mm/h (RR: 0-25) | Patchy heterogenous hypoechoic areas | NA |
| 54 | SAT | * | NA | TSH 0.54 mIU/L (RR 0.38-5.33), fT4 13.42pmol/L (RR 7.86-14.41), Tg 41.1 (RR 1.15-50) anti-TPO Ab 5.8 IU/mL (RR 0-9), anti-TG Ab <0.9 IU/mL (RR 0-4), CRP 4.85 mg/L (RR 0-0.8), ESR 55 mm/h (RR: 0-25) | Patchy heterogenous hypoechoic areas | NA |
| 54 | SAT | * | NA | TSH 2.44 mIU/L (RR 0.38-5.33), fT4 11.8 pmol/L (RR 7.86-14.41), anti-TPO <0.25 IU/mL (RR 0-9), CRP 0.77 mg/L (RR 0-0.8), ESR 10 mm/h (RR: 0-25) | Patchy heterogenous hypoechoic areas in the right lobe | NA |
| 54 | SAT | * | NA | TSH 0.127 mIU/L (RR 0.38-5.33), fT4 11.40 pmol/L (RR 7.86-14.41), anti-TPO Ab 2.9 IU/mL (RR 0-9), anti-Tg Ab 7.9 IU/mL (RR 0-4), TRAb 0.9 IU/mL (RR <1.5), CRP 1.02 mg/L (RR 0-0.8), ESR 41 mm/h (RR: 0-25) | ill-defined heterogenous area in the right lobe 50x25 mm | NA |
| 54 | SAT | * | NA | TSH 4.44 mIU/L (RR 0.38-5.33), fT4 10.99 pmol/L (RR 7.86-14.41), anti-TPO Ab 1.2 IU/mL (RR 0-9), anti-TG Ab < 0.9 IU/mL (RR 0-4), TRAb NA IU/mL (RR <1.5), CRP 1.16 mg/L (RR 0-0.8), ESR 344 mm/h (RR: 0-25) | Patchy heterogenous hypoechoic areas | NA |
| 54 | SAT | * | NA | TSH 0.47 mIU/L (RR 0.38-5.33), fT4 19.11 pmol/L (RR 7.86-14.41), anti-TPO Ab 1.2 IU/mL (RR: 0-9), anti-Tg Ab 10.9 IU/mL (RR 0-4), TRAb <1.5 IU/mL (RR <1.5), CRP 10.5 mg/L (RR 0-0.8), ESR 53 mm/h (RR 0-25) | Patchy heterogenous hypoechoic areas, decreased vascularisation | NA |
| 54 | SAT | * | NA | TSH 0.018 mIU/L (RR 0.38-5.33), fT4 26.10 pmol/L (RR 7.86-14.41), anti-TPO Ab 4.1 IU/mL (RR 0-9), anti-Tg Ab <0.9 IU/mL (RR 0-4), TRAb <1.5 IU/mL (RR <1.5), CRP 0.3 mg/L (RR 0-0.8), ESR 44 mm/h (RR: 0-25) | Patchy heterogenous hypoechoic areas, decreased vascularisation | NA |
| 54 | SAT | * | NA | TSH <0.01 mIU/L (RR 0.38-5.33), fT4 51.48 pmol/L (RR 7.86-14.41), anti-TPO Ab 6.11 IU/mL (RR 0-9), anti-TG Ab <0.9 IU/mL (RR 0-4), TRAb 0.45 IU/mL (RR <1.5), CRP 13.63 mg/L (RR 0-0.8), ESR 55mm/h (RR: 0-25) | Patchy heterogenous hypoechoic areas | NA |
| 54 | SAT | * | NA | TSH 0.032 mIU/L (RR 0.38-5.33), fT4 12.23 pmol/L (RR 7.86-14.41), anti-TPO Ab 1.2 IU/mL (RR 0-9), anti-Tg Ab < 0.9 IU/mL (RR 0-4), CRP 1.9 mg/L (RR 0-0.8), ESR 42 mm/h (RR: 0-25) | Patchy heterogenous hypoechoic areas | NA |
| 54 | SAT | * | NA | TSH 0.01 mIU/L (RR 0.38-5.33), fT4 37.7 pmol/L (RR 7.86-14.41), anti-TPO Ab 28 IU/mL (RR 0-9), anti-Tg Ab 26 IU/mL (RR 0-4), TRAb 0.8 IU/mL (RR <1.5), CRP 42.9 mg/L (RR 0-0.8) | Patchy heterogenous hypoechoic areas, decreased vascularisation | low uptake |
| 54 | SAT | * | NA | TSH 0.6 mIU/L (RR 0.38-5.33), fT4 14.0 pmol/L (RR 7.86-14.41), CRP 5.2 mg/L (RR 0-0.8), ESR 33 mm/h (RR 0-25) | Patchy heterogenous hypoechoic area in the middle part of left lobe | NA |
| 54 | SAT | * | NA | TSH 0.43 mIU/L (RR 0.38-5.33), fT4 14.8 pmol/L (RR 7.86-14.41), anti-TPO Ab 0.7 IU/mL (RR 0-9), anti-Tg Ab <0.9 IU/mL (RR 0-4), CRP 1.70 mg/L (RR 0-0.8), ESR 60 mm/h (RR: 0-25) | Patchy heterogenous hypoechoic areas | NA |
| 54 | SAT | * | NA | TSH 0.03 mIU/L (RR 0.38-5.33), fT4 31.65 pmol/L (RR 7.86-14.41), anti-TPO Ab 1.2 IU/mL (RR 0-9), anti-Tg Ab < 0.9IU/mL (RR 0-4), TRAb 3 IU/mL (RR <1.5), CRP 0.6 mg/L (RR 0-0.8), ESR 18 mm/h (RR 0-25) | At SAT diagnosis : Patchy heterogenous hypoechoic areas, decreased vascularisation. At GD diagnosis : diffuse heterogenous thyroid gland | increased uptake |
| 54 | SAT | * | NA | TSH 0.038 mIU/L (RR 0.38-5.33), fT4 17.27 pmol/L (RR 7.86-14.41), anti-TPO Ab 4.7 IU/mL (RR 0-9), anti-TG Ab <0.9 IU/mL (RR 0-4), TRAb 0.82 IU/mL (RR <1.5), CRP 3.65 mg/L (RR 0-0.8), ESR 67 mm/h (RR: 0-25) | Patchy heterogenous hypoechoic areas, decreased vascularisation | NA |
| 54 | GD | * | NA | TSH < 0.015 mIU/L (RR 0.38-5.33), fT4 27.92 pmol/L (RR 7.86-14.41), anti-TPO Ab 195.7 IU/mL (RR 0-9), anti-Tg Ab 7.1 IU/mL (RR 0-4), TRAb 10.3 IU/mL (RR <1.5), CRP 0.84 mg/L (RR 0-0.8), ESR 11 mm/h (RR: 0-25) | Diffuse hyperplasia, increased vascularisation | increased uptake |
| 54 | GD | * | NA | TSH < 0.015 mIU/L (RR 0.38-5.33), fT4 12.15 pmol/L (RR 7.86-14.41), anti-TPO Ab 0.7 IU/mL (RR 0-9), anti-TG Ab <0.9 IU/mL (RR 0-4), TRAb 0.97 IU/mL (RR <1.5), CRP 0.8 mg/L (RR 0-0.8), ESR 18 mm/h (RR 0-25) | Diffuse hyperplasia, increased vascularisation | increased uptake |
| 54 | GD | * | NA | TSH 0.015 mIU/L (RR 0.38-5.33), fT4 33.1 pmol/L (RR 7.86-14.41), anti-TPO Ab 0.8 IU/mL (RR: 0-9), anti-Tg Ab 1.8 IU/mL (RR 0-4), TRAb 0.25 IU/mL (RR <1.5), CRP 0.6 mg/L (RR 0-0.8), ESR 17 mm/h (RR 0-25) | Diffuse hyperplasia, increased vascularisation | increased uptake |
| 54 | GD | * | NA | TSH 0.01 mIU/L (RR 0.38-5.33), fT4 25.5 pmol/L (RR 7.86-14.41), anti-TPO Ab 196 IU/mL (RR 0-9), anti-Tg Ab 167 IU/mL (RR 0-4), TRAb 1.9 IU/mL (RR <1.5), CRP 0.3 mg/L (RR 0-0.8), ESR 6 mm/h (RR: 0-25) | Diffuse hyperplasia, increased vascularisation | increased uptake |
| 39 | GD | Chest pain, dyspnea | NA | TSH 0.010 (RR 0.55-4.78), fT4 33.92 (RR 11.5-22.7), anti-TPO Ab 77.72 (<34), anti-Tg Ab 137.5 (<115), TRAb 6.42 (<1.75), ESR 5 (<20), CRP 0.05 (<5) | Diffuse hyperplasia, increased vascularisation | increased uptake |
| 39 | GD | Weight loss, dyspnea | NA | TSH <0.08 (RR 0.55-4.78), fT4 73.8 (RR 11.5-22.7), anti-TPOAb 41.03(RR <34), anti-TgAb NA, TRAb 6.3 (RR <1.75) | Increased vascularity | increased uptake |
| 39 | Recurrence of GD | Weight loss, palpitations | NA | TSH <0.008 (RR 0.55-4.78), fT4 26.61 (RR 11.5-22.7), anti-TPOAb NA, anti-TgAb NA, TRAb 4.24 (RR <1.75) | Increased vascularity | NA |
| 39 | SAT | Cervical pain | NA | TSH 0.113 (RR 0.55-4.78), fT4 31.4 (RR 11.5-22.7), anti-TPO Ab <15 (RR<34), TRAb <1.1 (<1.75), ESR 63 (<20), CRP 28.6 (<5) | Ill-defined hypoechoic lesions | low uptake |
| 39 | SAT | Cervical pain, fever | NA | TSH 0.012 (RR 0.55-4.78), fT4 94.73 (RR 11.5-22.7), anti-TPOAb <15 (RR <34), anti-TgAb 39.71 (RR <115), TRAb 1.41 (RR <1.75), ESR 85 (RR <20), CRP 34.65 (RR <5) | Ill-defined hypoechoic lesions | NA |
| 39 | GD + SAT | Cervical pain, fever | NA | TSH <0.012 (RR 0.55-4.78), fT4 36.98 (RR 11.5-22.7), anti-TPOAb <15 (RR <34), anti-TgAb 295.1 (RR <115), TRAb 2.9 (RR <1.75), ESR 74 (RR <20), CRP 36.51 (RR <5) | enlarged thyroid gland with ill-defined hypoechoic lesion in left | low uptake |
| 39 | PT + thyreotoxic periodic paralysis | Leg weekness | NA | TSH 0.012 (RR 0.55-4.78), fT4 37.39 (RR 11.5-22.7), anti-TPO Ab <15 (RR<34), anti-Tg Ab 203.3 (RR <115), TRAb < 1.1 (RR <1.75), ESR 37 (RR <20), CRP 5.16 (RR<5) | heterogeneous echogenicity, decreased vascularity | low uptake |
| 55 | GD | Anxiety, palpitations | Tachycardia | TSH 0.005 uIU/Ml, fT4 2.96 ng/dL (RR 0.6–1.12), TRAbs 7.98 IU/L (RR <2.9) | enlarged thyroid gland with pseudonodules, hypervascularization | NA |
| 55 | GD | Headache, nausea, asthenia, palpitations | Tachycardia, eye redness, superior palpebral retraction | TSH 0.004 uIU/Ml, fT4 4.96 ng/dL (RR 0.6–1.12), TRAbs 3.2 IU/L (RR <2.9) | enlarged, hypervascularisated thyroid gland | NA |
| 40 | GD | Fever, weight loss, asthenia | Goiter | TSH <0.004 mIU/L (RR 0.4–4.00), fT4 5.56 ng/dL (RR 0.7–1.7), TRAb 6.48 IU/L (RR 0–1.49), anti Tg Ab 30 IU/mL (RR 0-30), anti-TPO Ab 21 IU/mL (RR 0-10) | heterogeneous, enlarged thyroid gland with increased vascularization | NA |
| 56 | SAT | Cervical pain, palpitations, sweating | Tachycardia | TSH <0.01, fT4 4.58, TPOAb <28, ESR 62 | NA | NA |
| 42 | SAT | Cervical pain, fever | Goiter, thyroid tenderness, tachycardia | TSH <0.008 lU/mL (RR 0.4–4.2), fT4 1.92 ng/dL (RR 0.8–1.5), TRAb <1.10 U/L (RR 0–1.75), TSI <0.10 U/L (RR 0–0.55), anti-TPO Ab <0.5 U/mL (RR 0–5.6), Anti-Tg Ab 3.4 U/mL (RR 0–4.1) | asymmetrically enlarged, hypervascular heterogeneous right thyroid lobe | NA |
| 41 | GD | Nausea, vomiting, fatigue, insomnia, palpitations, distal tremor | Hyperreflexia, arhythmic heart sounds | TSH <0.001lgUi/mL (RR 0.27–4.4), fT4 3.57 ng/dL (RR 0.93–1.71), Anti-Tg Ab 210 Ui/mL(RR 0–44), Anti-TPO Ab 3405 Ui/mL(RR 0–5.6), TRAb 16.56 Ui/L (RR 0–1.75), TSI 380% (RR <140), CRP 2.2 mg/L (RR <7.44) | enlarged and hypervascularisated thyroid gland | NA |
| 41 | GD | Anxiety, insomnia, palpitations, distal tremor | NA | TSH <0.001 lgUi/mL (RR 0.27–4.4), fT4 1.84 ng/dL (RR 0.93–1.71), Anti-Tg Ab 33 Ui/mL (RR 0–44), Anti-TPO Ab 833 Ui/mL(RR 0–5.6),TRAb 5.85 Ui/L (RR 0–1.75), CRP 1.26 mg/L (RR <7.44) | NA | diffuse toxic goiter, cold nodule in the middle third of the right lobe, high radioactive tracer distribution in the rest of the gland |

Legend: NA: non available; RR: reference range ; TSH : thyroid stimulating hormone; fT4 free thyroxine; anti-TPO Ab: anti-thyroid peroxidase antibodies; anti-Tg Ab: anti-thyroglobulin antibodies; TRAb: thyrotropin receptor antibody; TSI: thyroid stimulating immunoglobulin; ESR: erythrocyte sedimentation rate; CRP: c-reactive protein; SAT: Subacute thyroiditis; GD: Graves' disease; PT: painless thyroiditis
