# Supplementary material for: Thyroid Inconveniences With Vaccination Against SARS-CoV-2: The Size of the Matter. A Systematic Review
Source: Front Endocrinol (Lausanne). 2022 Jun 23;13:900964. doi: 10.3389/fendo.2022.900964 (PMC9259875; doi:10.3389/fendo.2022.900964)
Supplement: Supplementary file 1 [file DataSheet_1.zip › Supplementary Table 3.DOCX]

| **Ref.**  **Supplemental Table 3. Vaccines and follow-up of patients** | **Diagnosis** | **Vaccine** | **Dose** | **Days until symptoms** | **Treatement** | **Follow up** |
| --- | --- | --- | --- | --- | --- | --- |
| 13 | GD | Pfizer-BioNTech | 2 | 37 | stop thyroxine replacement, carbimazole and propanolol | NA |
| 57 | SAT | Moderna | 1 | 8 | prednisone, propanolol ibuprofen | remission |
| 57 | PT | Pfizer-BioNTech | 1 | 10 | none, levothyroxine | After 8 weeks hypothyroidism (substituted) which persisted at 16 weeks (anti-TPO Ab positive) |
| 57 | GD | Pfizer-BioNTech | 1 | 12 | Methimazole | NA |
| 14 | SAT | Pfizer-BioNTech | 2 | 4 | prednisolone 30 mg/day for 2 days, propranolol LA 80 mg/day, then ibuprofen 400 mg 3x/day, short course of prednisolone | remission after 20 weeks |
| 43 | PT | Moderna | 1 | 7 | none | remission after 3 weeks |
| 43 | PT | Moderna | 1 | 7 | none | remission after 3 weeks |
| 44 | GD | Pfizer-BioNTech | 2 | 60 | methimazole | persistance |
| 44 | GD | Pfizer-BioNTech | 1 | 10-14 | methimazole | persistance |
| 44 | GD | Moderna | 1 | 10-14 | Methimazole | NA |
| 44 | GD | Pfizer-BioNTech | 1 | 50 | methimazole | NA |
| 44 | SAT | Moderna | 1 | 10-14 | FANS | after 6 weeks: subclinical hypothyroidism, no treatment |
| 44 | SAT | Moderna | 1 | 10-14 | FANS | after 4-5 weeks: subclinical hypothyroidism |
| 44 | SAT | Pfizer-BioNTech | 2 | 10-14 | FANS | remission after 4-5 weeks |
| 44 | GD + SAT | Pfizer-BioNTech | 1 | 10-14 | Methimazole and FANS | NA |
| 15 | SAT | Pfizer-BioNTech | 2 | 14 | FANS | remission after 12 weeks |
| 16 | SAT | AstraZeneca | 1 | 14 | ibuprofen 600 mg, then tapered prednisolone 50 mg/day | remission after 6 weeks |
| 16 | SAT | Moderna | 1 | 14 | symptomatic therapy with 600 mg ibuprofen daily, due to gastrointestinal intolerance, changement to diclofenac 50 mg/day, and symptoms improved after 2 weeks. | Stop treatment after 2 weeks, relapse at 4 weeks, still in follow-up |
| 17 | Aggravation of GD | CoronaVac (2 doses), AstraZeneca (3rd dose) | 3 | 4 | increase of methimazol to 5 mg/day | NA |
| 18 | SAT | Moderna | 1 | 15 | propranolol, ibuprofen, prednisone | NA |
| 18 | SAT | Pfizer-BioNTech | 1 | 10 | propranolol, ibuprofen | NA |
| 18 | Thyroiditis | Pfizer-BioNTech | 2 | 20 | diltiazem, ibuprofen | NA |
| 45 | SAT | Pfizer-BioNTech | 2 | 10 | paracetamol and ibuprofen at the beginning, then for aggravation of symptoms beginning of methylprednisolone (16 mg twice a day); no medication after the 1st episode | After 7 weeks clinical improvement, pathologic TFT with TSH 0.173 mUI/ml (RR 0.4–4) and normal fT4 |
| 19 | SAT | CoronaVac | 2 | 14 | Naproxen sodium 2×275mg and propranolol 2×20mg | After 4 weeks hypothyroidism and introduction of levothyroxine |
| 20 | SAT | Pfizer-BioNTech | 2 | 0,5 | ibuprofen and azithromycin, prednisolone (40mg daily reducing regime) and propranolol | remission after 4 weeks |
| 46 | SAT | AstraZeneca | 1 | NA | 40 mg/day propranolol | remission after 8 weeks |
| 21 | TED | Pfizer-BioNTech | 2 | 3 | intravenous teprotumumab | after the second dose, clinical improvement |
| 47 | SAT | Pfizer-BioNTech | 1 | 12 | Prednisolone | NA |
| 47 | SAT | Pfizer-BioNTech | 2 | 4 | Prednisolone | NA |
| 22 | SAT | CoronaVac | 2 | 4 | methylprednisolone 16 mg/day and propranolol 25 mg/12 hours | Clinical and TFT improving. After 4 weeks still under methylprednisolone 8 mg/day |
| 22 | SAT | CoronaVac | 1 | 4 | Methylprednisolone 16 mg/day and propranolol 25 mg twice a day | After 10 weeks still under 8 mg/d methylprednisolone |
| 22 | SAT | CoronaVac | 2 | 7 | no treatement | remission after 8 weeks |
| 48 | PT | Pfizer-BioNTech | 1 | 17 | no treatement | remission after 20 weeks |
| 48 | PT | Pfizer-BioNTech | 2 | 10 | no treatement | remission after 8 weeks |
| 23 | GD | Pfizer-BioNTech | 1 | 5 | methimazole 60 mg/day titrated up to 80 mg/day, propranolol 40 mg 3 times/day | Good control 8 weeks later under methimazole 80 mg/d |
| 23 | GD | Moderna | 1 | 7 | no treatement | After 6 months: TSH 0.01 µIU/mL, fT4: 21 pmol/l; the patient was asymptomatic and no treatment was introduced. |
| 23 | GD | Pfizer-BioNTech | 2 | 28 | methimazole 5 mg/day, atenolol 25 mg/day | After 6 weeks: TSH still suppressed, symptomatology improved |
| 24 | Hypothyroidism and myxoedema | Pfizer-BioNTech | 2 | 21 | 14.2 µg/day, increased by 7.1 µg/day every 5 days up to 100 µg/day. Betamethasone tapered off over 2 weeks | clinical improvement |
| 25 | SAT | Covaxin | 1 | 5-7 | oral prednisolone 15 mg/day, taper of 5 mg every 2 weeks, for a total of 6 weeks. Oral propranolol 20 mg, twice/day discontinued after 2 weeks | remission after 7 weeks |
| 49 | GD | Pfizer-BioNTech | 1 | 17 | Methimazole 20 mg/day, Propranolol 20 mg three times/day, and a 7- day course of Prednisone 20 mg/day | remission after 8 weeks |
| 50 | SAT | NA | 2 | 7 | NSAIDs and prednisone 40 mg/day for 7 days | follow-up planned 2 months |
| 26 | SAT | Pfizer-BioNTech | 1 | 4 | Methylprednisolone 16 mg/day | remission after 8 weeks |
| 26 | SAT | AstraZeneca | 1 | 21 | no treatement | remission after 8 weeks |
| 27 | Recurrence of GD | Pfizer-BioNTech | 2 | 10 | thiamazole 20 mg twice /day | NA |
| 51 | Recurrence of GD | Pfizer-BioNTech | 2 | 30 | Thyreostatic treatment | thyreostatic treatment led to a rapid normalization of thyroid function |
| 51 | GD | Pfizer-BioNTech | 1 | 15 | Thyreostatic treatment | Thyreostatic treatment led to a rapid normalization of thyroid function |
| 28 | GD | Pfizer-BioNTech | 1 | 6 | thiamazole, potassium iodine, corticosteroid, furosemide and carvedilol | remission after 3 weeks |
| 29 | SAT | CoronaVac | 2 | 19 | ibuprofen | remission |
| 30 | SAT | Pfizer-BioNTech | 1 | 6 | NA | NA |
| 31 | GD | Pfizer-BioNTech | 2 | 14 | methimazole 20mg two times/day, atenolol 25mg | normalisation of TFT under treatement, developpent of TED 10 weeks later |
| 32 | SAT | Pfizer-BioNTech | 2 | 8 | acetylsalicylic acid 500 mg/6 hours and propranolol 20 mg/12 h | remission |
| 32 | SAT | Pfizer-BioNTech | 2 | 6 | (before admission to the clinic: Naproxen sodium 550 mg/8 h and propranolol 20 mg/12 hours) Acetylsalicylic acid 500 mg/6 hours and propranolol 20 mg/12 hours | Improving of symptoms on the second week, control scheduled after one month |
| 32 | SAT | Pfizer-BioNTech | 1 | 4 | Ibuprofen 600 mg/8 hours | control scheduled after 2 weeks |
| 32 | SAT | Coronavac (2 doses), Pfizer-BioNTech (3rd dose) | 3 | 6 | Acetylsalicylic acid 500 mg/6 hours and propranolol 20 mg/12 hours | remission after 4 weeks |
| 32 | SAT | Pfizer-BioNTech | 2 | 9 | Ibuprofen 600 mg/8 hours | remission after several weeks |
| 33 | SAT | Moderna | 2 | 5-6 | Analgesics (NSAIDS-no further specification) as needed | 8 weeks later asymptomatic and mild hypothyroidism |
| 34 | SAT | AstraZeneca | 1 | 21 | Propranolol, over-the-counter ibuprofen and paracetamol | 6 weeks later severe hypothyroidism (positive anti-TG) and introduction of levothyroxine |
| 35 | GD | AstraZeneca | 1 | 5 | carbimazole | NA |
| 35 | SAT | Pfizer-BioNTech | 1 | 4 | NA | Clinical improvement after 2 weeks |
| 36 | SAT | Pfizer-BioNTech | 2 | 3 | naproxen | remission after 4 weeks |
| 36 | SAT | AstraZeneca | 1 | 1 | ibuprofen and propranolol | After 2 months asymptomatic and mild hypothyroidism. New evaluation is scheduled. |
| 37 | SAT | Cansino Biologics | 1 | 15 | NSAIDs | At 4 weeks: asymptomatic, hypothyroidism, for the moment she is in follow-up without treatment |
| 38 | SAT + bilateral optic neuritis | CoronaVac | 2 | 0,5 | 1 g of methylprednisolone/day for 5 days | After 3 days of endovenous corticosteroid therapy normalisation of TSH, with tapering oral corticosteroid therapy improvement of ophtalmologic status. |
| 52 | SAT | Coronavac | 1 | 6 | Ibuprofen 1,200 mg/day, propranolol 20 mg twice a day | remission |
| 52 | SAT | Pfizer-BioNTech | 1 | 3 | methylprednisolone 16 mg/day and propranolol 20 mg twice a day | After 3 weeks clinical and TFT improvement, even if still pathologic: TSH 0.07, fT3 3.02 |
| 53 | SAT | Moderna | 1 | 5 | Prednisone 30 mg tapered on 40 days | remission after 11 weeks |
| 54 | SAT | Pfizer-BioNTech | 1 | 4 | NSAID 1 week | remission after 14 weeks |
| 54 | SAT | CoronaVac | 2 | 1 | prednisolone 5 mg/day, then 10 mg/days, tapered down and discontinued after 4 weeks | remission after 5 weeks |
| 54 | SAT | Pfizer-BioNTech | 1 | 10 | occasional paracetamol | remission after 13 weeks |
| 54 | SAT | CoronaVac (2 doses) and Pfizer-BioNTech (2 doses) | 2 | 15 | none | remission after 5 weeks |
| 54 | SAT | CoronaVac | 1 | 1 | NSAID 2 weeks | remission after 6 weeks |
| 54 | SAT | CoronaVac | 2 | 15 | metilprednisolone 16 mg/day, tapered down and discontinued after 4 weeks | remission after 20 weeks |
| 54 | SAT | CoronaVac | 2 | 4 | metilprednisolone 16 mg/day, tapered down and discontinued after 4 weeks | not in remission |
| 54 | SAT | CoronaVac | 2 | 7 | none | remission after 11 weeks |
| 54 | SAT | Pfizer-BioNTech | 1 | 10 | NSAID 2 weeks | remission after 4 weeks |
| 54 | SAT | CoronaVac | 1 | 13 | occasional paracetamol, NSAID 1 week during relapse | remission after 12 weeks |
| 54 | SAT | Pfizer-BioNTech | 2 | 7 | metilprednisolone 16 mg/day, tapered down and discontinued after 4 weeks | remission after 11 weeks |
| 54 | SAT | CoronaVac (2 doses), Pfizer-BioNTech (1 dose) | 3 | 3 | none | not in remission |
| 54 | SAT | Pfizer-BioNTech | 2 | 5 | NSAID --> metilprednisolone 16 mg/day, tapered down and discontinued after 4 weeks | remission after 18 weeks |
| 54 | SAT | CoronaVac | 1 | 4 | metilprednisolone 16 mg/day, tapered down and discontinued, 22 weeks --> methimazole 5 mg/day | not in remission |
| 54 | SAT | CoronaVac (2 doses), Pfizer-BioNTech (1 dose) | 3 | 10 | Prednisolone 20 mg/day | not in remission |
| 54 | GD | CoronaVac (2 doses), Pfizer-BioNTech (1 dose) | 3 | 2 | methimazole 10 mg/day | not in remission |
| 54 | GD | Pfizer-BioNTech | 1 | 15 | none | remission after 10 weeks |
| 54 | GD | CoronaVac (2 doses), Pfizer-BioNTech (1 dose) | 3 | 9 | methimazole 15 mg/day--> 10 mg/day | not in remission |
| 54 | GD | Pfizer-BioNTech | 1 | 14 | stop levothyroxine | hypothyroidism resurfaced ar 20th week |
| 39 | GD | AstraZeneca | 1 | 1 | NA | NA |
| 39 | GD | AstraZeneca | 2 | 14 | NA | NA |
| 39 | Recurrence of GD | Johnson & Johnson | 1 | 14 | NA | NA |
| 39 | SAT | AstraZeneca | 2 | 4 | NA | NA |
| 39 | SAT | AstraZeneca | 1 | 11 | NA | NA |
| 39 | GD + SAT | Johnson & Johnson | 1 | 14 | NA | NA |
| 39 | PT + thyreotoxic periodic paralysis | Johnson & Johnson | 1 | 10 | NA | NA |
| 55 | GD | AstraZeneca | 2 | 10 | propranolol 40 mg/day, thiamazole 15 mg/day --> rush--> propylthiouracil 150 mg/day | After 12 weeks good control under PTU 100 mg/d |
| 55 | GD | AstraZeneca | 1 | 5 | propranolol 20 mg/day, thiamazole 15 mg/day | After 12 weeks good control under methimazole 5 mg/d |
| 40 | GD | Pfizer-BioNTech | 2 | 28 | methimazole and atenolol | clinical improvement and normalization of TFT |
| 56 | SAT | Pfizer-BioNTech | 1 | 5 | prednisone 40mg/day, propranolol 20mg as needed | improvement |
| 42 | SAT | Pfizer-BioNTech | 2 | 13 | NSAID --> prednisone | NA |
| 41 | GD | Pfizer-BioNTech | NA | 2 | propranolol 60 mg, diltiazem 120 mg, ivabradine 5 mg, thiamazole 10 mg/day | NA |
| 41 | GD | Pfizer-BioNTech | NA | 3 | propranolol 40 mg/day, thiamazole 10 mg/day | NA |

**Supplemental Table 3. Vaccines and follow-up of patients**

Legend: NA: non available; TFT: thyroid function tests; TSH : thyroid stimulating hormone; fT4 free thyroxine; anti-TPO Ab: anti-thyroid peroxidase antibodies; anti-Tg Ab: anti-thyroglobulin antibodies; TRAb: thyrotropin receptor antibody; TSI: thyroid stimulating immunoglobulin; ESR: erythrocyte sedimentation rate; CRP: c-reactive protein; SAT: Subacute thyroiditis; GD: Graves' disease; PT: painless thyroiditis; NSAID: nonsteroidal anti-inflammatory drugs.
